# Optimization strategy of the emerging memristors: From material preparation to device applications

**DOI:** 10.1016/j.isci.2024.111327

**Published:** 2024-11-06

**Authors:** Kaiyun Gou, Yanran Li, Honglin Song, Rong Lu, Jie Jiang

**Affiliations:** 1 Hunan Key Laboratory of Nanophotonics and Devices, School of Physics, Central South University, Changsha, Hunan 410083, China; 2 State Key Laboratory of Precision Manufacturing for Extreme Service Performance, College of Mechanical and Electrical Engineering, Central South University, Changsha, Hunan 410083, China; 3 School of Electronic Information, Central South University, Changsha, Hunan 410083, China

**Keywords:** Physics, Engineering, Materials science

## Abstract

With the advent of the post-Moore era and the era of big data, advanced data storage and processing technology are in urgent demand to break the von Neumann bottleneck. Neuromorphic computing, which mimics the computational paradigms of the human brain, offers an efficient and energy-saving way to process large datasets in parallel. Memristor is an ideal architectural unit for constructing neuromorphic computing. It offers several advantages, including a simple structure, low power consumption, non-volatility, and easy large-scale integration. The hardware-based neural network using a large-scale cross array of memristors is considered to be a potential scheme for realizing the next-generation neuromorphic computing. The performance of these devices is a key to constructing the expansive memristor arrays. Herein, this paper provides a comprehensive review of current strategies for enhancing the performance of memristors, focusing on the electronic materials and device structures. Firstly, it examines current device fabrication techniques. Subsequently, it deeply analyzes methods to improve both the performance of individual memristor and the overall performance of device array from a material and structural perspectives. Finally, it summarizes the applications and prospects of memristors in neuromorphic computing and multimodal sensing. It aims at providing an insightful guide for developing the brain-like high computer chip.

## Graphical abstract

**Figure:**
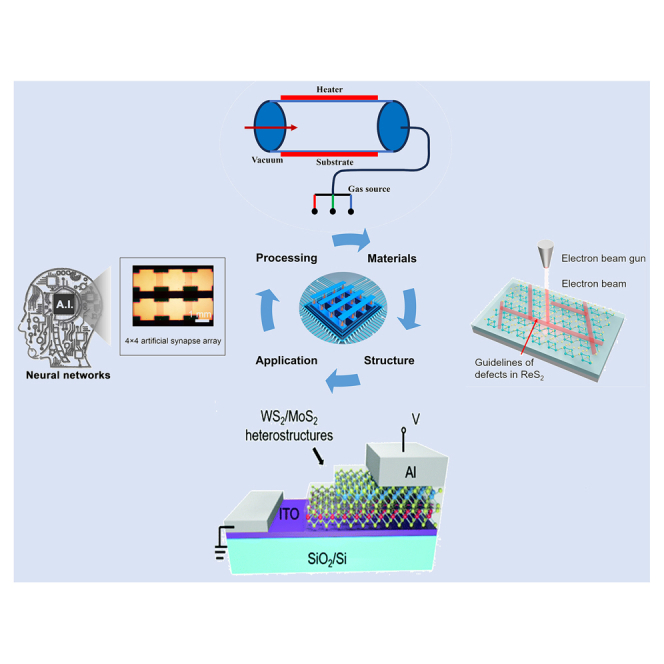

## Introduction

Moore’s law predicts that the number of transistors on a chip doubles every two years, boosting performance and complexity.^1^^,^^2^ With the continuous progress of the manufacturing process, the size of the transistor continue to shrink, and the calculating speed and functional complexity of the chip have been greatly improved. At present, the 5-nanometer process (the smallest characteristic size of transistors) becomes the main technology of top-of-line chip manufacturing. What’s more, some foundry companies begin to launch the 3nm process, such as Samsung and TSMC. As chip technology gets smaller, it brings many challenges, such as: quantum effects, thermal effects, cost, and complexity issues. At the same time, with the rapid development of artificial intelligence (AI) and the Internet of Things (IOT), higher requirements are put forward for the data processing capabilities of devices.^3^^,^^4^^,^^5^^,^^6^ However, the traditional von Neumann computing architecture, with its separate storage and processing units, leads to serious issues of power consumption and signal delay.^7^^,^^8^ Thus, enhancing traditional computing power by process alone is limited. We must innovate at the architectural level to overcome von Neumann bottleneck, providing more efficient and energy-efficient solutions for large data processing.

At present, many emerging technologies, such as heterogeneous computing, memory computing, neuromorphic computing, quantum computing, optical computing, edge computing, etc.,^9^^,^^10^^,^^11^^,^^12^^,^^13^ are used to explore new computing architectures. Among them, neuromorphic computing is a new computing paradigm inspired by the human brain, which has the characteristics of high energy efficiency, low power consumption, parallel, and autonomous learning, etc.^14^ The human-brain neural network built through synapses and neurons can improve computing power and reduce power consumption, which is a potential candidate for a new computing architecture. As early as 2014, IBM of the United States built the brain-like chip True North by integrating 5.4 billion silicon transistors, and the computing power and performance of the chip showed exponential growth and low power consumption.^15^ However, the True North chip is a hardware network based on traditional silicon-based transistors, and the complexity of the process has also increased exponentially. In fact, early in 1971, Leon Chua predicted the passive basic component (“memristor”) lost in the fourth from the completeness of circuit theory.^16^ It is the fourth basic circuit element after resistance, capacitance, and inductance. In 2008, HP Laboratories first established the link between the memristor theory and experimental results, making the memristor move from theory to practice.^17^ The memristor is a type of circuit element characterized by its simple structure, low power consumption, small size, and fast response speed. The resistance value of the memristor varies in response to the level of charge or voltage passing through it. When the power supply is disconnected, it can retain this resistance value until it is altered by a subsequent passage of charge or voltage. Therefore, it is considered to be an ideal unit for the future neuromorphic computation.

**Figure 1:**
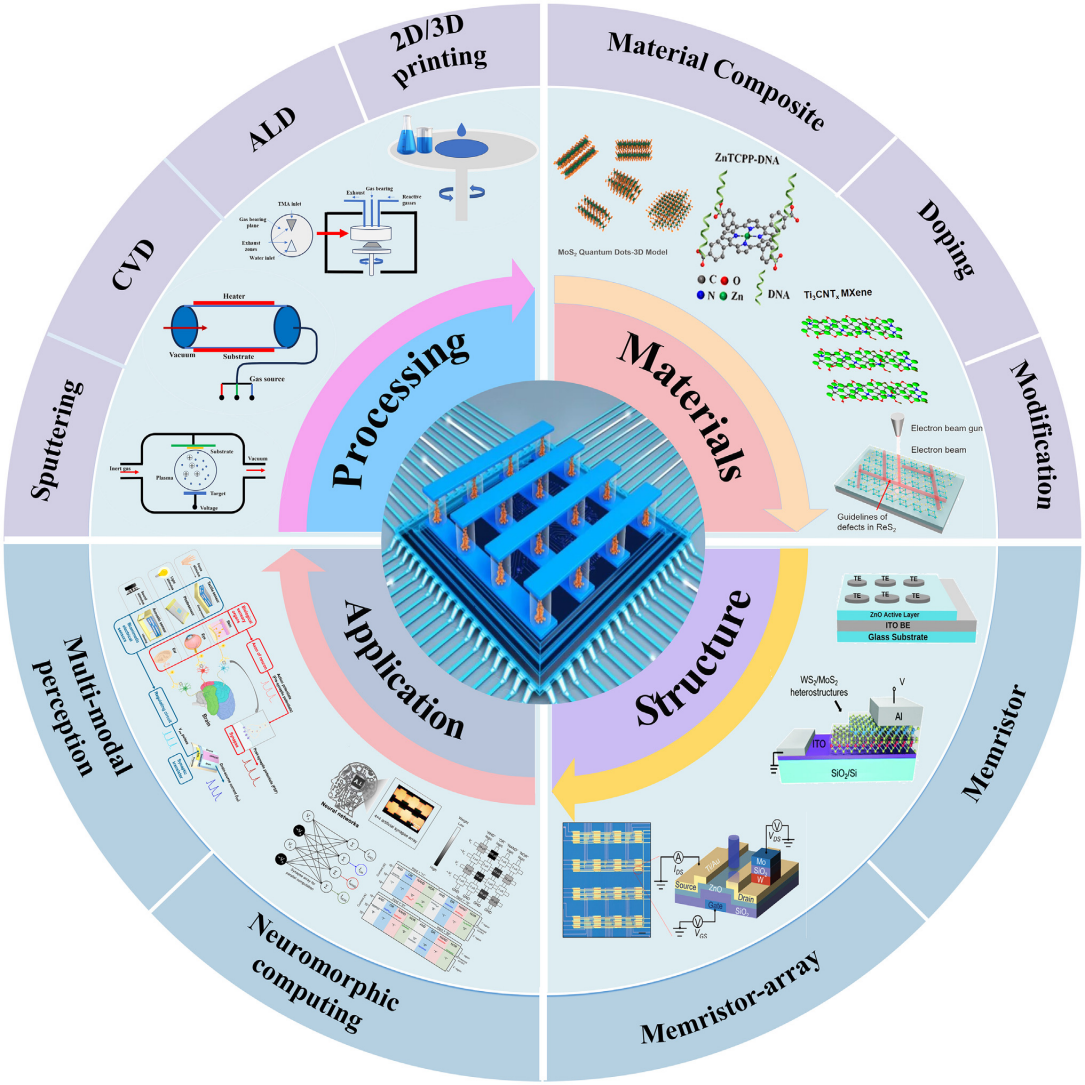
The main content of this review: Optimization strategy from device fabrication to application The figures in materials section are reproduced with permission from Ref.^27^ Copyright © 2023 American Chemical Society. Ref.^28^ Copyright © 2023 American Chemical Society. Ref.^29^ Copyright © 2024 American Chemical Society. Ref.^30^ Copyright © 2021, Springer Nature. The figures in structure section are reproduced with permission from ref.^31^ Copyright © 2022, Elsevier Ltd. Ref.^32^ Copyright © 2021, Royal Society of Chemistry. Ref.^33^ Copyright © 2022 Wiley-VCH GmbH. The figures in application section are reproduced with permission from ref.^34^ Copyright © 2022 Wiley-VCH Verlag. Ref.^35^ Copyright © 2023, UESTC and John Wiley & Sons Australia, Ltd.

Since the concept of memristor was proposed, many new materials and structures of memristor have been proposed,^18^^,^^19^^,^^20^^,^^21^ such as metal oxides, two-dimensional materials, organic materials, heterojunction, etc. After this, further exploration is carried out on the application of devices and arrays, such as image recognition, multi-modal perception, construction of hardware-like brain systems, and three-dimensional integration, etc.^22^^,^^23^^,^^24^^,^^25^^,^^26^ First of all, the device performance is an unavoidable topic for the possible device applications. In neuromorphic devices, resistive materials and device structures determine the performance of the memristor, such as switching ratio, endurance, retention, switch speed, and resistance switching (RS). In this review, we are focusing more on strategy to improve the performance of the device, as shown in Figure 1. Firstly, the common fabrication methods of memristors are described, including sputtering, chemical vapor deposition (CVD), atomic layer deposition (ALD) and 2D/3D printing, and the effects of the parameters and gas environment on the microstructure of the material during the preparation are analyzed. Next, the effects on device performance will be discussed from the perspective of material composite and modification. Then, the optimization methods of devices and arrays are summarized from the structure. Finally, the newest applications of memristor in multi-sensory simulation, neuromorphic computing, and memory storage are introduced. At the end of this paper, the current challenges of memristor are summarized and prospected.

## Optimization strategy based on device fabrication

So far, various semiconductor materials, such as metal oxides, two-dimensional materials, organic materials, perovskite material, etc.,^36^^,^^37^^,^^38^ have been explored and the switching layer of the memristor has been successfully constructed.^39^ Therefore, selecting the best fabrication technology for materials is the key to determining device performance. At present, the fabrication methods can be roughly divided into physical deposition and chemical deposition in principle, including sputtering, CVD, ALD, 2D/3D printing. These are mostly adopted in large-area fabrication.^40^ For example, the fabrication of metal oxides mostly uses sputtering^41^ and ALD.^42^ Two-dimensional materials mostly use CVD. Of course, in the patterning fabrication process, 2D/3D printing has better design flexibility. In this section, the effects of large area preparation techniques on materials, such as crystallinity, defects, microstructure, etc., will be discussed in device.

### Chemical vapor deposition

**Figure 2:**
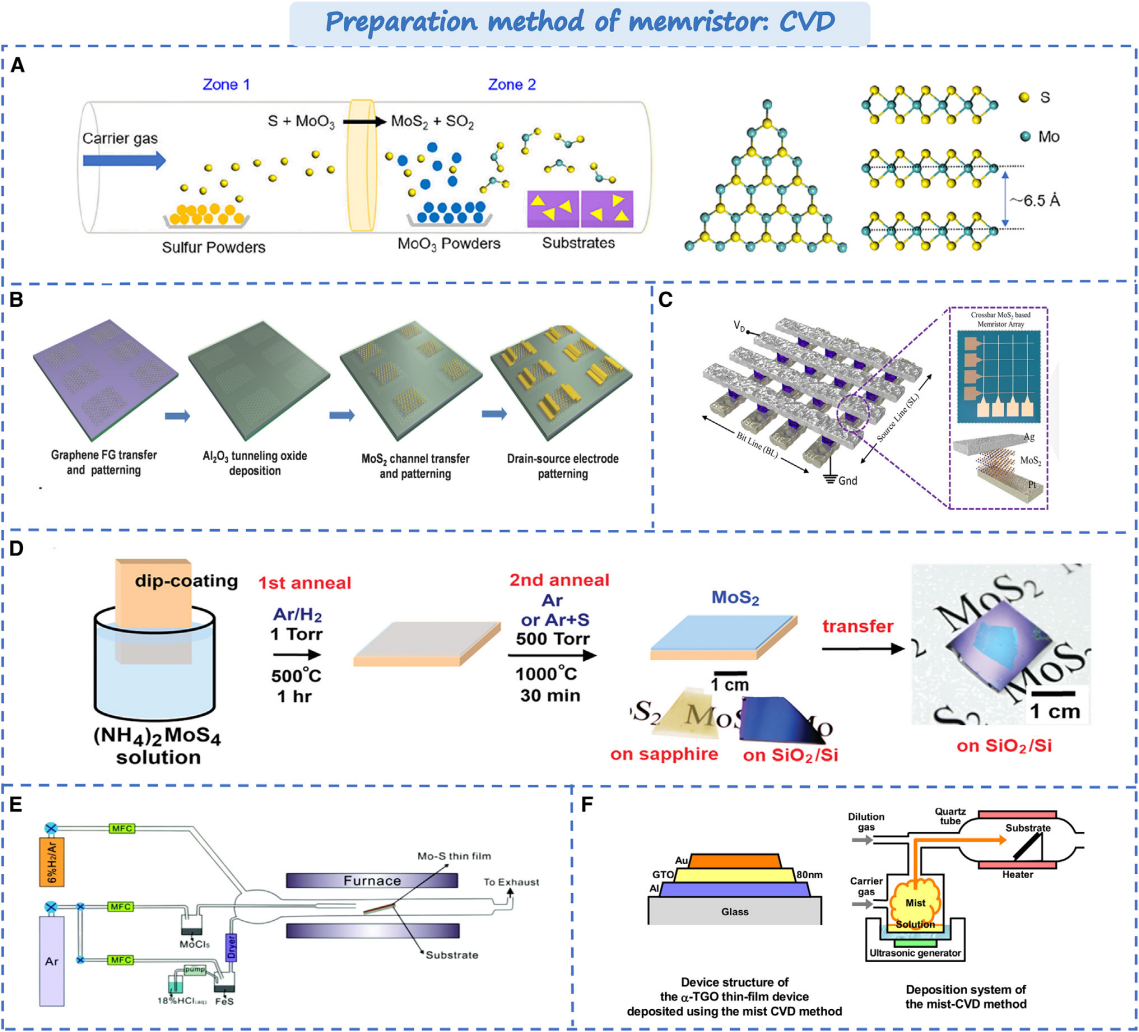
Preparation method of memristor based on CVD (A) Direct synthesis of monolayer MoS_2_ single crystals by CVD.^44^ Copyright © 2021, American Chemical Society. (B) Fabrication process for an array by large-area CVD-grown.^45^ © 2017 WILEY-VCH Verlag GmbH & Co. KGaA, Weinheim. (C) The large-area direct grown MoS_2_ array.^46^ Copyright © 2022, Springer Nature. (D) The two-step thermolysis process of MoS_2_ thin layers.^48^ Copyright © 2012 American Chemical Society. (E) The fabrication of APCVD apparatus.^50^ Copyright © 2022, Royal Society of Chemistry. (F) The mist CVD method and deposition system of the mist-CVD method for the α-TGO thin-film.^51^ Copyright © 2020, AIP Advances.

Chemical vapor deposition (CVD) is a technology that uses gaseous or vapor substances to chemically react on a solid surface to generate solid deposits under certain temperature and pressure conditions. CVD plays a crucial role in modern materials science and manufacturing.^43^ It allows for precise control over the composition, structure, and thickness of the film, resulting in specific electrical, chemical, and mechanical properties. Zhao et al. utilized CVD to fabricate a single layer of molybdenum sulfide (MoS_2_),^44^ as shown in Figure 2A. The single-layer MoS_2_, as a direct band gap material, exhibits more sensitive optical response characteristic and demonstrates superior on/off state and tunability in devices. Additionally, CVD has been widely employed in the fabrication of the large-area films. As shown in Figure 2B, Yu et al. employed CVD to grow large-area two-dimensional layered memristor arrays.^45^ The device array shows outstanding uniformity with an average switching ratio of 10^4^ and over 8,000 cycles memory durability. Furthermore, through the combination of sputtering and CVD technology, Kim et al. fabricated a 4 × 4 crossbar array, as shown in Figure 2C. Its durability up to 500 cycles and retention time up to ∼10^4^s.^46^ The resistance state is highly homogeneous, indicating significant potential for memristor fabrication based on CVD technology. In addition, several CVD-based variants, such as low-pressure CVD, atmospheric pressure CVD (APCVD), and plasma-enhanced CVD (PECVD) have been utilized for the fabrication of high-quality films. By controlling the environment in which the film is deposited, the preparation of high-quality films can be achieved. Yoo et al. employed plasma-enhanced chemical vapor deposition technology to synthesize 7–10 nm MoS_2_ films.^47^ Plasma activation can enhance the activity of chemical gas phase reaction substances, increase the surface reaction rate, and significantly reduce the deposition temperature of thin films by high-energy ions. It enables a more uniform, high-quality film deposition. Furthermore, Li et al. and W. Hawak et al. enhanced the crystallinity and electrical properties of molybdenum disulfide using PECVD and APCVD combined with two annealing processes,^48^^,^^49^ as shown in Figure 2D. In contrast to PECVD, Hao et al. proposed utilizing salt-assisted CVD for preparing two-dimensional layered WSe_2_ nanosheets,^50^ as shown in Figure 2E. It shows the good scalability of CVD technology. Additionally, Kimura et al. introduced an atomized vapor deposition method,^51^ in Figure 2F. The Al electrode is oxidized in the oil mist CVD process, and the AlOx layer is formed on the film surface of the bottom terminal. The AlOx layer blocks the drift and diffusion of oxygen ions. Thus, the stability of the resistive. Amorphous Sn-Ga-O (α-TGO) thin film devices deposited via hot-wall fog-CVD can be manufactured on the large area at low temperature and cost-effectively provide new perspectives for future high-performance device preparation.

### Atomic layer epitaxy

**Figure 3:**
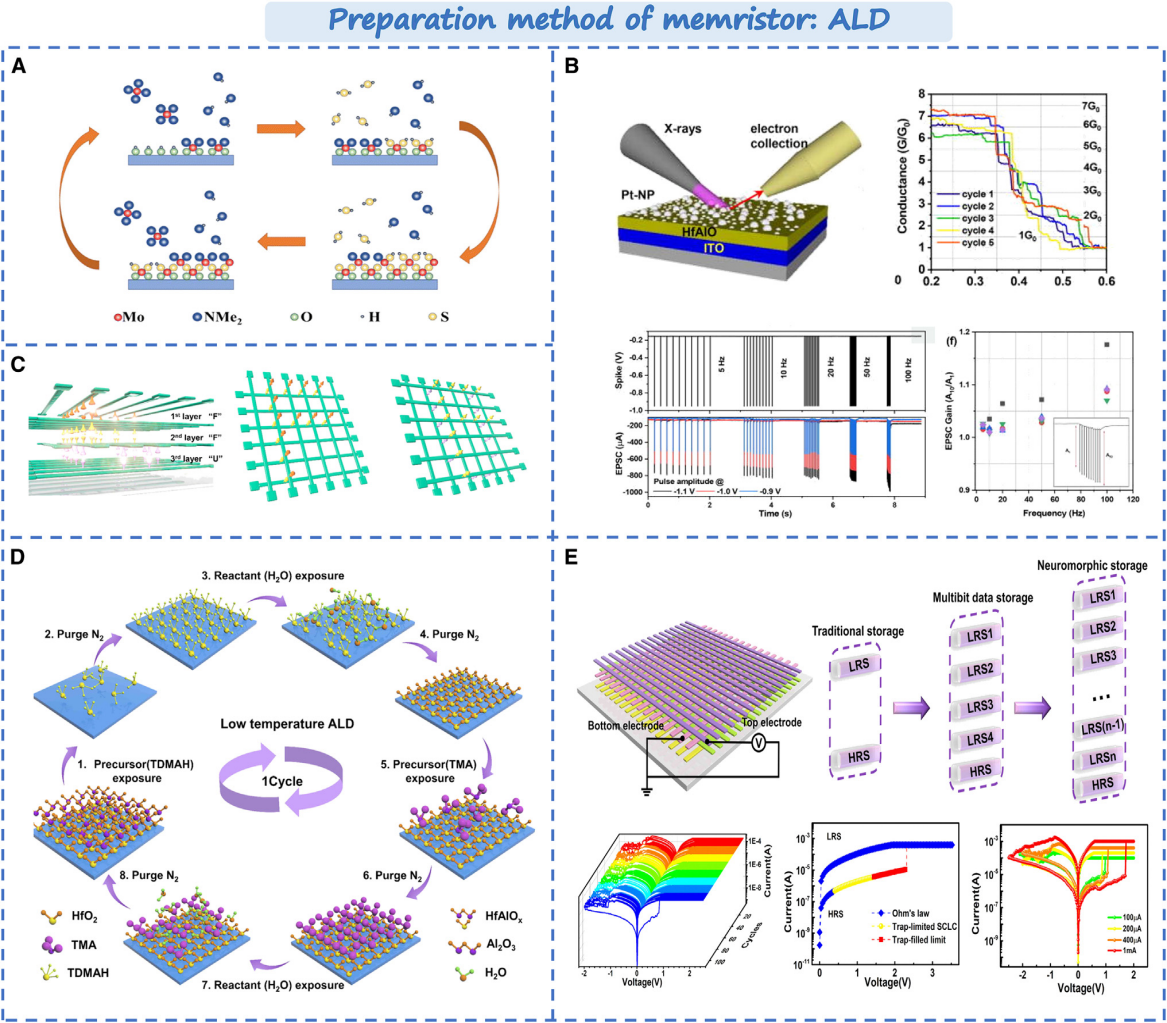
Preparation method of memristor based on ALD (A) ALD growth process of the MoS_2_ film.^55^ Copyright © 2023, Royal Society of Chemistry. (B) Pt-nanoparticle incorporated HfAlO_x_ alloy memristor conductance modulation characteristics.^56^ Copyright © 2021, AIP Publishing. (C) The 3D neural network of synaptic units.^57^ Copyright © 2020, American Chemical Society. (D) Low temperature ALD process based oxide (HfAlO_x_) devices. (E) The ability of multibit data storage.^58^ Copyright © 2020, John Wiley & Sons Australia.

Atomic layer deposition (ALD) is a technique used to manufacture ultra-thin films that deposit material layer by layer by chemical reaction by alternately introducing different chemical vapor phase precursors on the substrate surface. Whereas ALD can achieve layer by layer deposition of materials, it can realize smaller device and high-density integration. ALD offers the excellent material applicability, three-dimensional conformal property, and high step coverage rate.^52^^,^^53^^,^^54^ It has widespread applications in various fields, such as microelectronics manufacturing, biocatalysis, energy storage etc. Li et al. utilized atomic layer deposition to fabricate based MoS_2_ memristor with high linear resistance.^55^ As shown in Figure 3A, the amorphous MoS_2_ film can be transformed into the polycrystalline film through the atomic-scale control and annealing treatment. The device can avoid the dependence of random grain boundaries. Kim et al. deposited Pt nanoparticles between the hafnium aluminum oxide (HfAlO_x_) switching layers,^56^ as shown in Figure 3B. It leads substantial improvements in conductance modulation characteristics, such as frequency and amplitude dependence. Furthermore, for heat-sensitive materials like plastics and polymers, the device performance can be affected by thermal diffusion during the preparation process. Low temperature atomic layer deposition can avoid this issue without cracks, defects or pinholes. In addition, ALD has many advantages in 3D structure deposition, such as better step coverage rate, 3D conformal property, regional selectivity, and low thermal budget. Zhang et al. developed an artificial intelligence system with efficient information integration and computing capabilities using low temperature ALD,^57^ as shown in Figure 3C and its deposition process as shown in Figure 3D. The three-dimensional cross-bar array has multistage information transmission function, power consumption is 4.28aJ and response speed is 50ns. In the same year, Zhang et al. developed a three-dimensional flexible memristor array by low temperature atomic layer deposition.^58^ As shown in Figure 3E, the device achieves multi-bit storage, and has a long retention time. Compared with traditional structure, the resistance state of the vertically stacked memristor network shows an exponential increase. It proves the importance of ALD for 3D device stacking and makes an important step for the future development of ultra-efficient, ultra-high-speed wearable 3D neuromorphic computing systems.

### Sputtering

**Figure 4:**
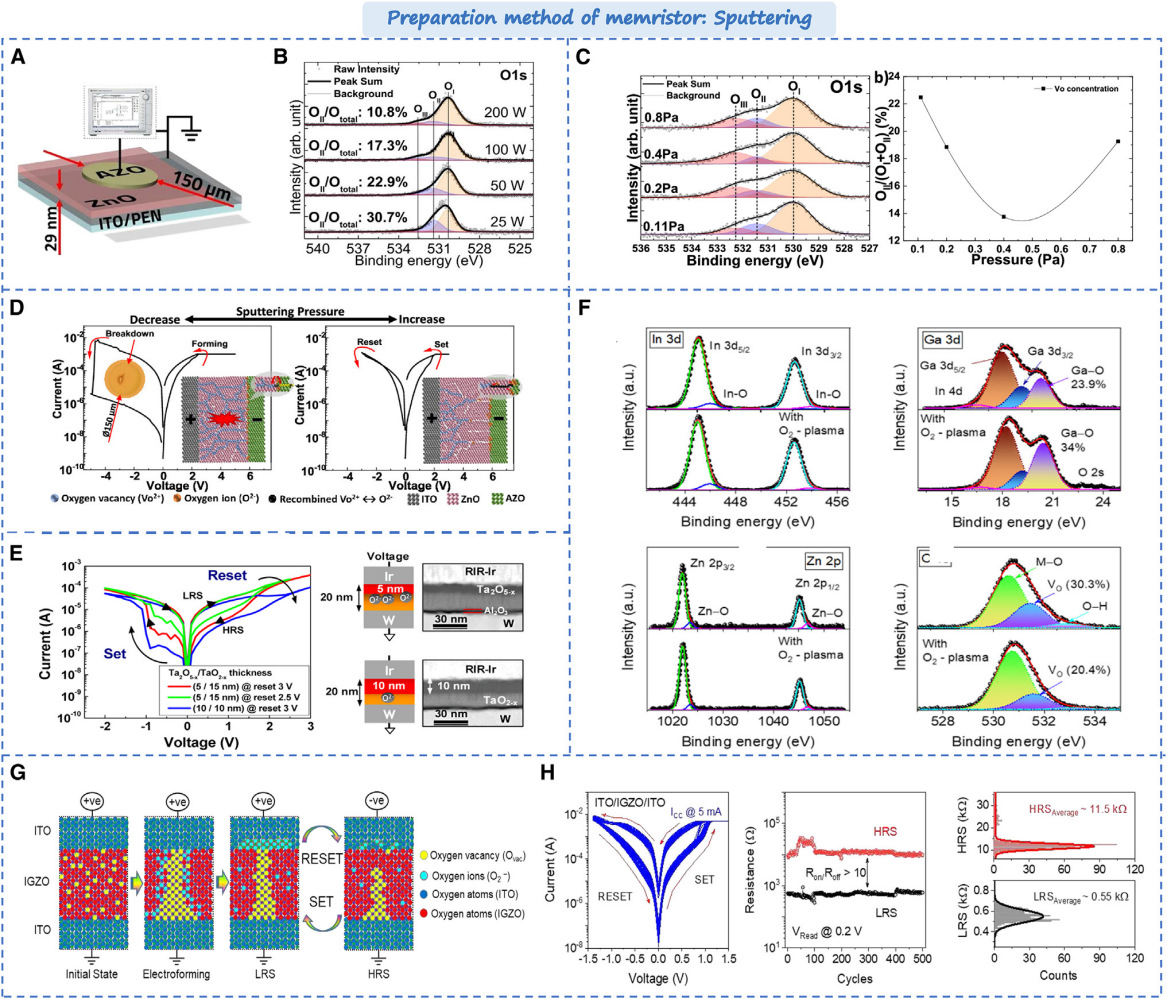
Preparation method of memristor based on Sputtering (A) Schematic of the Zno device. (B) The oxygen content of ZnO films at different RF powers.^61^ Copyright © 2019, AIP Advances. (C) The oxygen-vacancy concentration in ZnO films at different sputtering pressures. (D) I-V curves of devices under different pressures as well as setup and reset.^62^ Copyright © 2019, American Chemical Society. (E) The different set/reset process in various Ta_2_O_5–x_/TaO_2–x_ thickness. (F) The RF deposited InGaZnO resistive switching layer without and with O_2_-plasma treatment. (G) Switching mechanism with a schematic description of O^−^ and V_O_ movements. (H) Bipolar resistive switching characteristics of the ITO/IGZO/ITO memristor with O_2_ plasma treatment.^63^ Copyright © 2019, Journal of Chemical Physics.

Sputtering is a physical vapor deposition (PVD) technique used to prepare thin film materials. Sputtering technology uses high-energy particles (usually argon ions) to bombard the target material (target) in a high vacuum environment, so that the atoms or molecules on the surface of the target are sputtered out and deposited on the substrate to form a thin film. It has the characteristics of fast speed, low temperature and little damage to the film.^59^ In oxides-based memristors, the controlling of oxygen vacancy concentration is crucial for device performance. Kim et al. prepared the ZnO based memristor using radio frequency sputtering.^60^ Through high-resolution transmission electron microscopy (TEM), the formation of oxygen vacancy defects was observed in polycrystalline ZnO during the reaction of Zn and O_2_, which confirmed that the oxygen vacancies were directly involved in the formation process of conductive filaments. Ohno et al. investigated the influence of RF power and vacuum degree on the performance of ZnO memristor during sputtering.^61^^,^^62^ The device structure is shown in Figure 4A, and the XPS characterization of oxygen vacancy concentration in different condition is shown in Figures 4B and 4C. The oxygen vacancy concentration is low under high power, and the device exhibits excellent resistance characteristics and uniformity. As the RF power decreases, the switching behavior becomes poor and may breakdown the device. However, under the high vacuum, the oxygen vacancy concentration tends to decrease, and the devices have WORM (Write Once, Read Many) storage characteristics, as shown in Figure 4D. Under higher vacuum, the oxygen vacancy concentration will increase. The devices show the reproducible switching characteristics, while they show ineffable data storage characteristics in low pressure deposition process. In addition, the oxygen vacancy concentration can also be improved by plasma treatment during sputtering. The energy of the plasma is transferred to the surrounding region, facilitating the interaction between the plasma and the target material. Thus, the quality of the film sputtering is enhanced. Kim et al. controlled oxygen vacancy concentration through oxygen plasma,^63^ as shown in Figure 4E. As oxygen is inlet during sputtering, oxygen vacancies in indium gallium zinc oxide (IGZO) films significantly decrease. As shown in Figures 4F and 4G, the memristor showed better stability during the formation of conductive filaments. At the same time, some researchers have proposed some new sputtering techniques, such as magnetron co-sputtering process and dual ion-beam sputtering,^64^^,^^65^ which provides new insights for the construction of high-performance devices in the future.

### 2D/3D printing

**Figure 5:**
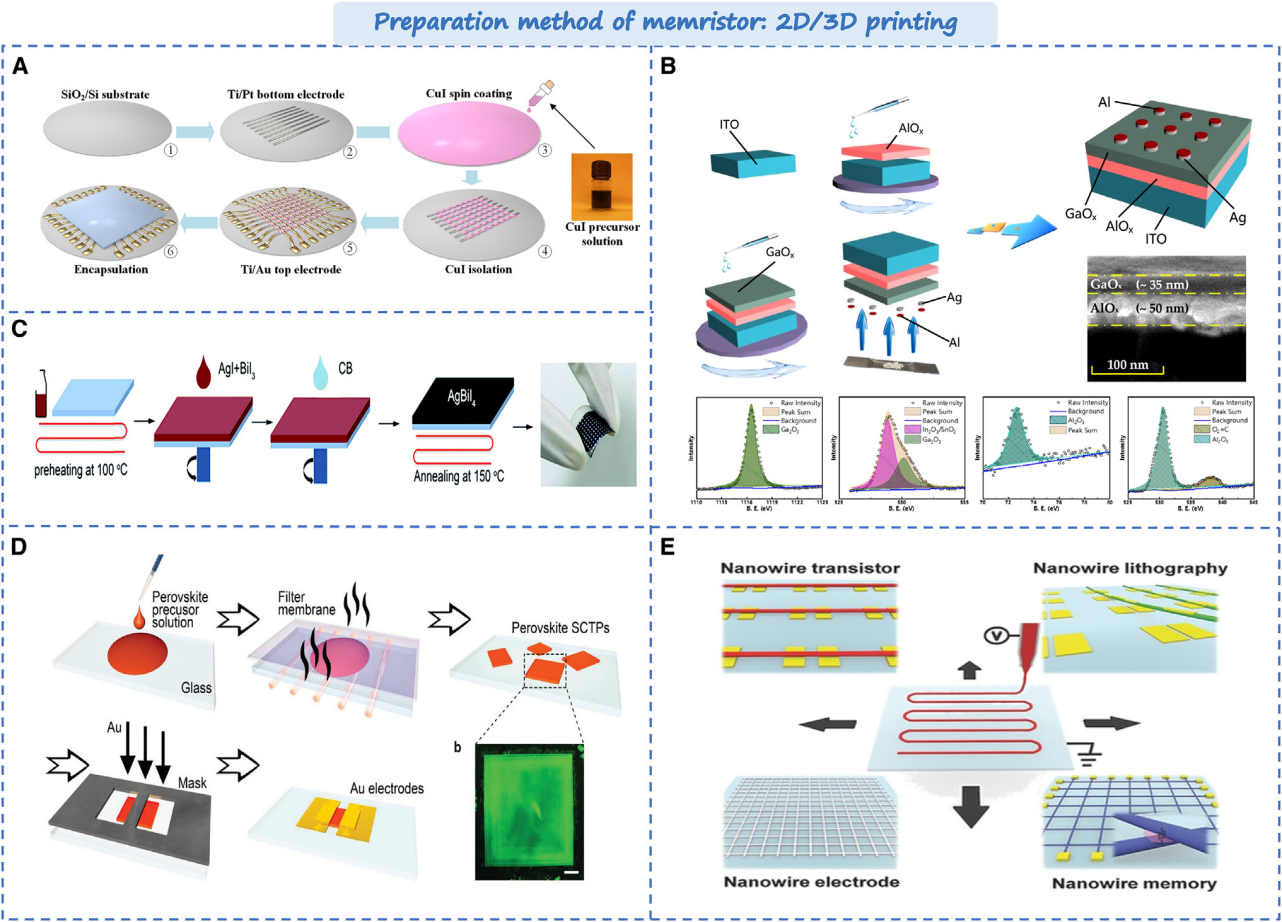
Preparation method of memristor based on 2D/3D printing (A) The fabrication process for the Pt/CuI/Cu memristor array.^69^ Copyright © 2024, Elsevier Ltd. (B) The fabrication process of Ag/SP-GaO_x_/SP-AlO_x_/ITO RRAM devices.^70^ Copyright © 2021, American Chemical Society. (C) Schematic illustration for fabricating AgBiI_4_ layers, including a hot-coating process and a post-annealing process at 150°C.^71^ Copyright © 2020, Royal Society of Chemistry. (D) Synthesis and characterization of MAPbBr_3_ SCTPs.^72^ Copyright © 2020 Wiley-VCH GmbH. (E) The schematic of e-nanowire printing.^78^ Copyright © 2017 WILEY-VCH Verlag GmbH & Co. KGaA, Weinheim.

The 2D/3D printing technology can achieve the simple, uniform, and rapid preparation of functional materials.^66^^,^^67^^,^^68^ Compared to complex vacuum deposition or lithography techniques, 2D/3D printing technology is easier to operate. It can prevent high-temperature damage to materials and flexible substrates. Currently, according to the dimension of material, 2D/3D printing like spin-coating, drop-casting, and 3D printing are wide-established and extensively used in device fabrication. Its operation is simple, and the film can be prepared under normal pressure and room temperature conditions. Zhu et al. made a Pt/Cul/Pt memristor array using spin coating and laser etching, as shown in Figure 5A.^69^ The new synaptic devices provide reliable performance in switching speed and matrix calculations. In order to further enhance the uniformity and crystallinity of the film, as shown in Figure 5B, Zhao et al. fabricated the device by spinning coating.^70^ By controlling the rotation speed, a higher-quality and uniform film can be fabricated. Liao et al. proposed the dynamic hot casting approach to enhance the crystallinity of the material.^71^ As shown in Figure 5C, during the spinning period, the hot perovskite precursor is drop-casted onto the heated substrate, accelerating solvent evaporation and forming uniform, pinhole-free grains with outstanding crystallinity. In addition, the solution-treated material possesses certain flexibility. As shown in Figure 5D, Xu’s co-workers reported a surfactant-assisted self-assembly approach for fabricating based methylammonium bromide lead bromide (MAPbBr_3_) single crystal sheet.^72^ The thickness and size of the sheet can be regulated by this method, and the device features an ultra-low working current. It offers a novel scheme for future synaptic devices in neuromorphic bioelectronics. However, spin-coating has limit in fabricating patterned films.^73^ 3D printing technology holds distinctive advantages in thin film patterning, such as inkjet printing, aerosol printing, electrohydrodynamic (EHD) printing, screen printing, and hybrid printing.^74^^,^^75^^,^^76^ In the printing process, the solution concentration is a crucial factor for device performance. Solanki et al. achieved two-dimensional perovskites with different layers by controlling the concentration of the screen-printing solution.^77^ In addition, the 3D printing technology enables the complete printing-based fabrication of the device ranging from electrode to channel, thereby significantly reducing the processing complexity. As shown in Figure 5E, EHD printing can precisely control the thickness and pattern of the nanowire deposition.^78^ By freely adjusting the orientation and position of the alignment, accurately printing of the pattern is achieved. The performance of the device is guaranteed while the patterned printing is realized. Similarly, the deposition quality of the film is ensured by controlling the environment in which it is prepared. Such as low-temperature aerosol jet printing^79^ and low-temperature inkjet printing with thermal annealing and DUV treatment.^80^ It opens up a new way for the preparation of devices. Bermak et al. reviewed the process parameters, systems, materials, and other requirements for additive manufacturing in memristor fabrication.^81^

## Optimization strategy based on memristor materials

As the deeper research on device in recent years, numerous new resistive switching (RS) materials have emerged, such as two-dimensional materials, metal oxides, and organic materials.^82^^,^^83^^,^^84^ These new materials offer new possibilities for the high performance and new functions of devices. Nevertheless, most materials enhance the performance of devices only unilaterally or in several aspects. It is not enough to realize the new architecture computing paradigm of storage and computing. To further enhance the key characteristic of memristor like the storage window, switching speed, retention time, endurance, and power consumption. Modification or improvement of the composition, microstructure, surface, and size of the material to meet the needs of different applications is necessary.^85^ In this section, we first review the emerging materials based on memristors and summarize their advantages and disadvantages. Finally, some new optimization methods are proposed to improve the performance of memristors. The effects on device performance will be discussed from the perspective of material composite and modification of materials.

### New RS materials based on memristors

#### Metal oxides

**Figure 6:**
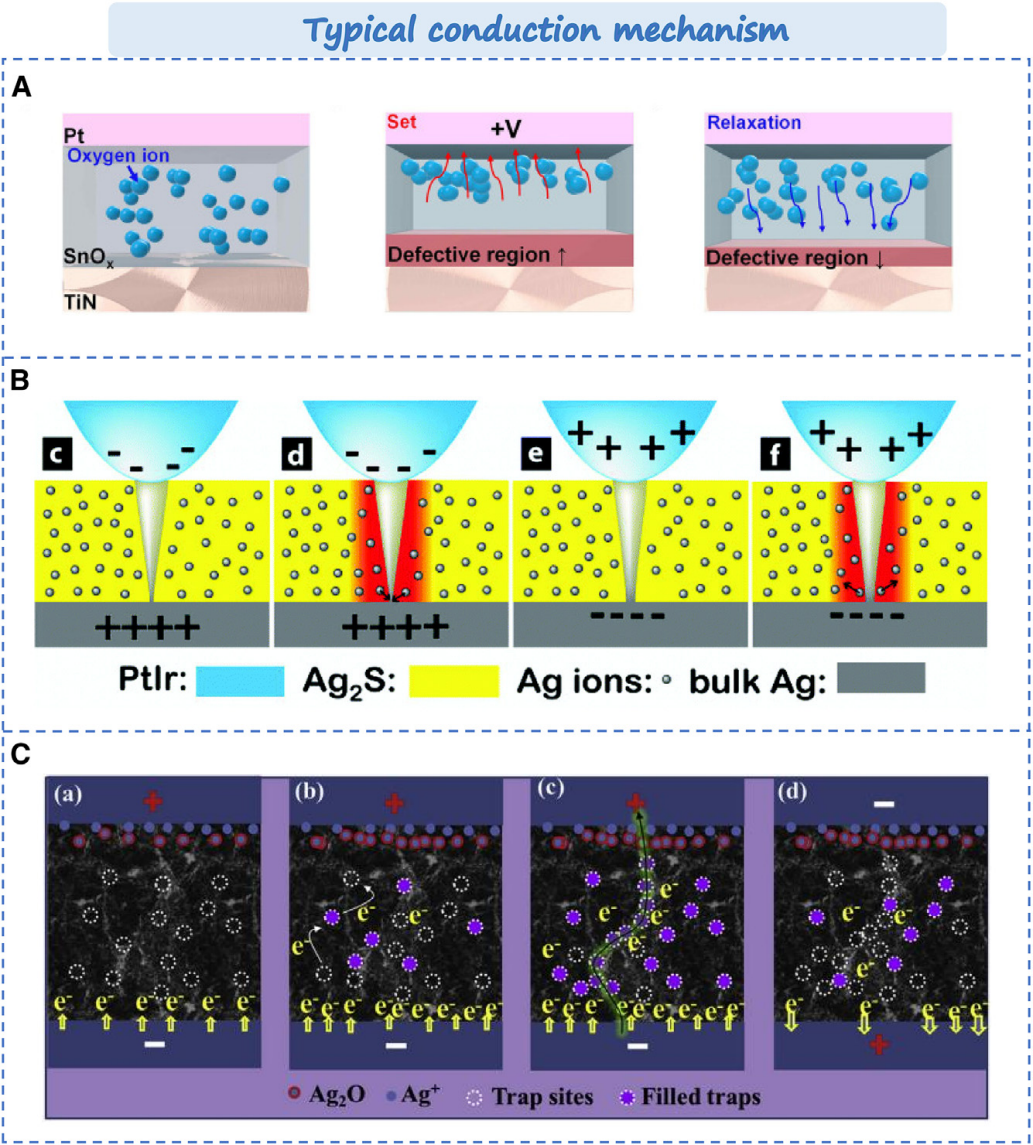
Typical conduction mechanism based on memristor (A) Conduction mechanism based on oxygen vacancy.^86^ Copyright © 2024, Elsevier. (B) Thermal assisted phase transition conduction mechanism.^91^ Copyright © 2024, Elsevier. (C) Physical models of charge trapping and detrapping processes.^92^ © 2019 Published by Elsevier Ltd.

In recent years, metal oxides have been widely used in RS layer of memristors owing to their excellent electrical and optical properties. In metal oxide based memristors, except for conductive filaments based on metal cations, there is also a kind of conductive mechanism dominated by oxygen vacancy. Jin et al. reported the Pt/SnO_x_/TiN memristor.^86^ Its conductive filaments are generated through the migration of oxygen ions. As shown in Figure 6A, the non-filamentous switching mechanism induces the migration of oxygen ions under applied bias voltage, leading to the formation of defect regions. When a positive bias is applied to the Pt electrode, oxygen ions in SnO_x_ move toward the top electrode, thereby increasing defect areas and facilitating filament formation. It will result in resistance switching from high resistance state (HRS) to low resistance state (LRS). Conversely, during a gradual reduction in applied bias, self-diffusion of oxygen ions occurs, diminishing defect areas and promoting insulating behavior that leads to resistance switching from LRS back to HRS. Currently, metal oxides can be primarily categorized into binary metal oxides and polymetallic oxides such as SiO_x_, TiO_x_, AlO_x_, ZnO_x_, SrTiO_3_, SrZrO_3_, LaCa_1-x_MnO_3_. Due to their straightforward structure and compatibility with conventional CMOS processes, binary metal oxides are most been selected in memristor materials, including TiO_x_, AlO_x_, NiO_x_, CuO_x_, ZnO_x_, HfO_x_, TaO_x_, WO_x_, ZrO_x_, SnO_x_, etc.^87^ Notably, HfO_x_^88^ and TaO^89^ exhibit significant potential owing to their sub-nanosecond operational speeds and durability cycles exceeding 10^10^. However, memristors based on binary metal oxides typically suffer from high power consumption and low uniformity in large-scale array fabrication. Additionally, polyoxides like ITO and IGZO are considered promising materials for developing next-generation artificial vision systems due to their exceptional light-responsive characteristics. For example, developed by Zhuge’s team achieved reversible modulation of its conductive state by varying the wavelength of incident light signals in IGZO based memristor.^90^ Although IGZO memristors offer considerable advantages, such as low power consumption, high resolution and all-optical regulation. Their reversibility stability and technological maturity regarding all-optical control still remain challenging.

#### Chalcogenides material

Chalcogenides consist of metallic elements and chalcogenide elements (S, Se, Te, etc.). Most chalcogenide compounds demonstrate high ionic mobility, which is beneficial for the formation and fracture of conductive filaments in memristors. RS phenomena have been observed in various chalcogenide materials. These devices exhibit excellent performance characteristics including GeS, GeSe, MoS_2_, WS_2_, MoTe_2_, MoSe_2_, etc. A variety of conduction mechanisms have been confirmed in chionogen-based memristors including conductive filament formation, atomic vacancy migration, electron capture, and release. For example, Golberg et al. reported the Ag/Ag_2_S/W memristor.^93^ The RS mechanism based on Ag conducting filaments was confirmed by high-resolution transmission electron microscopy. This mechanism has also been confirmed in other structures, such as Ag/Ag_2_S_3_:Ag/Au, Ag/GeSe:Ag/Ni, Pt:Ir/GeS:Cu/Pt:Ir.^94^^,^^95^^,^^96^ In addition, Jin et al. confirmed the resistance switching behavior induced by phase transition in Cu_2_Se material.^91^ The resistance switching (RS) mechanism is based on the transformation of phase change materials from an amorphous to a crystalline state to realize resistance switching. As shown in Figure 6B, the degree of phase transition of the material is controlled by the Joule thermal effect. When the heat exceeds the crystallization temperature, crystallization in the amorphous region will be caused. On the contrary, it will gradually change to an amorphous state. Furthermore, Mihaly et al. demonstrated that overheating results in a transition of Ag_2_S material and leads to a switch in resistance values within a memristor based on this material.^97^ Moreover, with the thicknesses of materials (TMDs) decrease, it mostly appears in two-dimensional form. It has ultra-thin dimensions and excellent photoelectric properties which make them competitive materials with potential for mitigating Moore’s Law. For example, as the size of MoS_2_ decreases (<10nm), its band gap changes significantly. Its light absorption ability and electron mobility is obviously improved, which can significantly improve the switching speed of the device and reduce power consumption. In addition to chalcogenide compounds, such as graphene, boron nitride (BN), 2D erovskites, InSe, SnS, TiS_2_, black phosphorus (BP), MXenes, etc., have also emerged.^98^ Among them, MXenes material has been the hot materials owing its simple preparation process and ultra-thin thickness. It is an effective way to achieve high-density device integration. Despite the obvious advantages of 2D materials, due to the small thickness of 2D materials, it also brings some challenges, such as: large area preparation, stability around the atmosphere, biocompatibility, etc.

#### Ferroelectric materials

Ferroelectric materials can realize reversible polarization transition under the influence of an electric field, and possess characteristics, such as spontaneous polarization, piezoelectricity, and pyroelectricity. In contrast to traditional memristors, ferroelectric memristors alter the polarization state of ferroelectric materials to realize resistance switching. Currently, many memristors based on ferroelectric materials have demonstrated outstanding device performance. For example BiFeO_3_ and BaTiO_3_ . As reported by Yan et al., the switching ratio of the memristor based on Hf_0.5_Zr_0.5_O_2_ can reach 10^4^.^99^ The Ag/BaTiO_3_/Nb:SrTiO_3_ memristor can operate at a maximum speed of 600 ps and a 5-bit number of states.^100^ Furthermore, sub nanosecond resistance switches maintain up to 358 K with a write current density as low as 4 × 10^3^ A cm^−2^. It shows the potential for ferroelectric materials to achieve ultra-fast, low-power, and high-density non-volatile memory capabilities. In addition, III-N semiconductors have gotten significant attention in the development of new generation semiconductor materials.^101^ After doping with rare earth elements such as Nb or Sc, it will acquire their wide and adjustable band gap, high electron mobility, chemical stability properties along with large spontaneous polarization properties. The ferroelectricity and superconductivity of the material can be enhanced. For example. The introduction of Sc can alter the lattice constant and lattice structure of AlN, thereby affecting the ferroelectric phase transition temperature. Simultaneously, hybridization between the 3d electrons of Sc and the 2p electrons of N can strengthen spontaneous polarization. Furthermore, Sc doping has the potential to increase both saturation polarization and coercive field strength of AlN. Additionally, metal Sc doping can improve the structural stability and rigidity of AlN materials, enabling them to maintain favorable ferroelectric properties in high temperatures and harsh environments. Moreover, the preparation technology for ScAlN is compatible with CMOS technology, making it an ideal material for future ferroelectric memristors. However, at present, except for some emerging materials, traditional single ferroelectric material memristors often underperform in terms of durability and retention time compared to expectations.

#### Carbon-based materials

Carbon-based materials exhibit various forms and structures, such as zero-dimensional carbon quantum dots, fullerenes, one-dimensional carbon fibers, carbon nanotubes, carbon nanowires, two-dimensional graphene, and three-dimensional bulk materials. Currently, many carbon-based materials are used as the switching layer material for memristors and possess excellent physical, chemical, electrical, and thermal properties. For example, Scott et al. reported the durability of the device based on Al/GOAu/ITO can reach 10^2^, and the retention time can reach 10^3^.^102^ In addition, Song et al. realized self-powered device based on memristor and amorphous carbon-based nanogenerator using only carbon-based materials.^92^ The memristor based on carbon-based materials presents a physical model involving charge trapping and detrapping processes. As shown in Figure 6C, it shows defects formed by O-C bonds, C-atom vacancies and distortions. When no electric field is applied, the trap sites are randomly distributed in the switching layer, and some traps are filled with injected charges as the electric field is applied. It will cause the output current to gradually increase. As the applied voltage increases, all traps are filled. This leads to the formation of conductive filaments, causing the memristor to switch from HRS to LRS. A decapture process occurs when the scan direction is reversed. Shi et al. reported that the Pt/a-C:H/TiN memristor durability can reach 10^7^, retention time up to 10^4^.^103^ The mechanism of resistance switching in carbon-based materials is elucidated based on the hydroredox model. The conductive mechanism of LRS is attributed to the formation of conjugated double bonds by dehydrogenation of the conductive sp^2^ carbon filament, while the conductive mechanism of HRS is attributed to the formation of an insulating sp^3^ carbon filament. It provides a new idea for high performance devices based on new materials and new mechanisms.

#### Polymer materials

Polymer materials have excellent stability, durability, and biocompatibility. Compared with metal oxides, sulfides, amorphous silicon and other inorganic counterparts, polymer materials have unique characteristics, such as low cost, easy processing, mechanical flexibility and ductility. Furthermore, the electronic properties of polymer materials can be adjusted through molecular design, and synthesis strategies. They have attracted widespread attention in information storage and neuromorphic applications. In the field of polymer resistors, various mechanisms based on polymeric materials have been proposed successively, such as charge transfer, phase change, conformational changes, and oxidation-reduction mechanisms. Currently, various materials have been used in memristor including PEDOT: PSS, PMMA, PFN/PBS, PFcFE, PA-1, etc.^104^ These show excellent stability, durability, low variability, and outstanding flexibility. Wong et al. reported the non-volatile memristors based on ferrocene polyfluorenyl acetylene.^105^ PFcFE1, PFcFE2, PFcFE3, and PFcFE4 based memristors exhibit non-volatile resistance switching behavior and have “WORM” storage function. In addition to multi-storage information, the device also showed high ON/OFF current ratio from 10^3^ to 10^4^, low conductive voltage (-1V), long retention time (10^3^s), and large read cycles (10^5^). Furthermore, by adjusting the chemical structure of the main chain, the storage characteristics of the polymers can be finely tuned. Some degradable biomaterials exhibit unique properties in “green electronics”, such as proteins and their composites, sugars and deoxyribonucleic acid (DNA).^106^ However, the device performance is unstable in biomaterial memristor. Li et al. reported a memristor based on Ag-doped chitosan.^107^ In undoped memristor, the size and dimensions of conductive filament formed are random, resulting in unstable switch performance. The instability problem is improved by doping Ag^+^. For biological materials, memristor have important implications for practical applications.

In conclusion, with the successive emergence of various materials and the continuous exploration of device functions, it is often insufficient to rely solely on the performance of a single material for practical applications. For example, the working power of ferroelectric materials and the stability of polymer materials. Therefore, specific methods are needed to further optimize the performance of devices. In next section, we will focus on elaborating specific optimization methods based on memristor materials from the perspective of memristor mechanism, such as material composite, material doping, material modification, and reconstruction.

### Typical modification methods based on memristor materials

#### Materials composite

**Figure 7:**
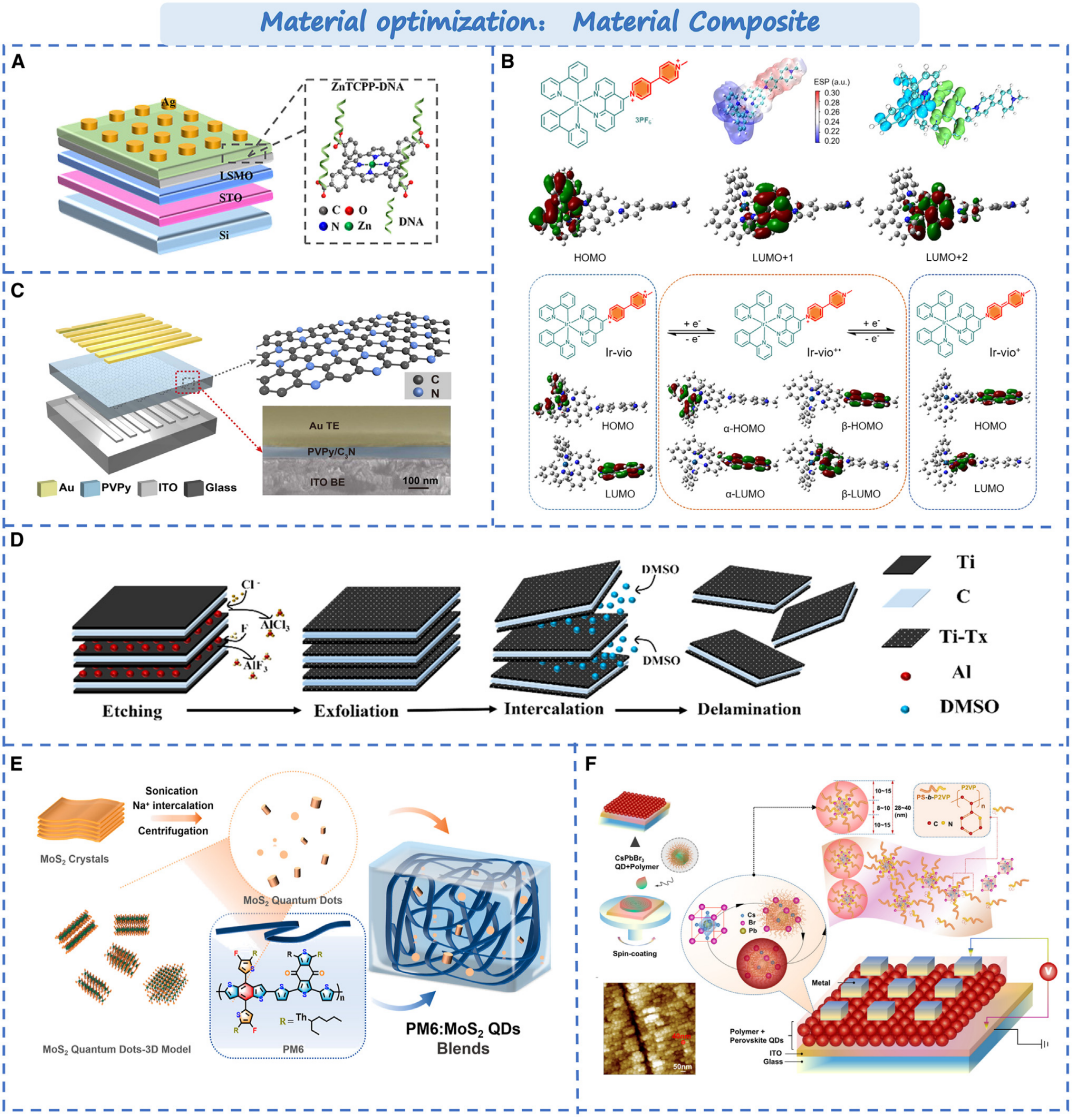
Optimization strategy based on memristor by material composite (A) The ZnTCPP–DNA-based device structure.^27^ Copyright © 2023, American Chemical Society. (B) The complex Ir-vio in different redox states.^110^ Copyright © 2021, American Chemical Society. (C) The C_3_N/PVPy-based memristor structure.^111^ Copyright © 2019, Elsevier Ltd. (D) The fabrication process of 2D Ti_3_C_2_T_x_ MXene and delamination.^112^ Copyright © 2024, American Chemical Society. (E) Preparation of MoS_2_ QDs and the Blended System with PM6 in CHCl_3_ Solution.^28^ Copyright © 2023, American Chemical Society. (F) The CsPbBr_3_ QD-based resistive random-access memory (RRAM) device.^116^ Copyright © 2023, American Chemical Society.

As is well known, biological materials can offer good biocompatibility and stability but lack in electronic mobility, switching control, processing speed, stability, and scalability.^108^^,^^109^ However, when it is combined with organic or inorganic materials, device performance significantly improves while retaining their unique properties. Yan et al. Combined DNA with Zinc (II) Tetrakis (4-carboxyphenyl) porphyrin (ZnTCPP) to create the composite membrane,^27^ as shown in Figure 7A. DNA is a complex large molecule that contains nucleotides, carbohydrates, and phosphate groups. It can provide a good channel for the formation of CF. The switching ratio, power consumption, cycle stability, and data retention greatly improved compared to single based ZnTCPP device. Zhao et al. added the porphyrin fragment to its N^∧^N ligand to form the electroactive iridium (III) complex Ir-vio,^110^ as shown in Figure 7B. The surface of Ir-vio molecules has continuous positive electrostatic potential along the conjugate skeleton, which can provide charge transfer channels, and the interconversion between different REDOX states of Ir-vio can achieve polymorphic memristor behavior. The devices show multilevel storage characteristics, high switching ratios, and low threshold voltages. In addition, organic polymer materials exhibit excellent biocompatibility, flexibility, and transparency. For example, polydimethylsiloxane (PDMS), polyvinyl alcohol (PVA), and polyethylene oxide (PEO) are commonly used in various applications. Han et al. compounded two-dimensional C_3_N with the polyethylene pyrrolidone (PVPy) material, forming a uniform hydrogen bond network between the C_3_N and PVPy,^111^ as shown in Figure 7C. Through simulating the generation and release of neurotransmitters via similar proton dynamics. Since the surface diffusion energy barrier of PVPy is relatively large, it is only when the voltage bias is increased to a certain extent. Protons can overcome the surface diffusion barrier of PVPy and diffuse to C_3_N nanosheets. Thus the switch of resistance state is realized. The synaptic characteristics and stability of the device can be improved. Similarly, such as PDMS with graphite,^112^ PVA and graphene oxide,^113^ acrylonitrile (NNA), and N-vinyl carbazole (PVK),^114^ In addition, composite with metal oxides can change the type of metal ions that form the conductive filaments. The performance of the device can get significantly improved. As shown in Figure 7D, Solanki et al. controlled the retention of aluminum metal ions during the etching of Ti_3_C_2_T_x_ MXene.^115^ The introduction of multiple quantum well structures results in multiple high energy barriers in Ti_3_C_2_T_x_. The retained aluminum ions lower the energy barrier between p-Ti_3_C_2_T_x_, reduce the operating voltage, and decrease the power consumption of the device.

In addition to composite with some compounds, quantum dots are often used in material compounding owing to their good stability, biocompatibility, versatility, and self-assembly ability. Zhang et al. resolved the inherent microstructural inhomogeneity of the polymer film by combining MoS_2_ quantum dots with polymer films.^28^ As shown in Figure 7E, molybdenum disulfide quantum dots offer the active center for the conductive channel through electrons capture and detrapping. The directional formation of the conductive channel between PM6 and MO2QD is controlled and randomness is reduced, providing a narrow switching voltage range and cycle life for the device. In addition, The CD-based memristors also shows the outstanding performance, such as graphene quantum dots (GQDs)^117^ and carbon dots. Zhou et al. reported an based Zr_0.5_Hf_0.5_O_2_:GOQDs memristor with bidirectional tunability, low power consumption, and rapid switching characteristics.^118^ Subsequently, Choi et al. reported a type of organic nitrogen-doped graphene oxide quantum dots memristor.^119^ The device demonstrates ion migration dynamics similar to the biological synapses, providing a solution for future biocompatible neural systems. Besides the carbon nanodots, perovskite quantum dots and multicomponent quantum dots are also extensively utilized in devices. As shown in Figure 7F, Xiong et al. synthesized core-shell nanosphere composites using CsPbBr_3_ quantum dots and block copolymer polystyrene—polystyrene—vinyl pyridine.^116^ The enhanced electric field of S_2_VP-CSPBBR_3_QDS composite membrane can drive Br—ions to the core-shell interface near the Al anode more effectively. At the same time, more Al^3+^ is repelled to the ITO cathode and reduced to the Al cluster. The spontaneous formation of S_2_VP-CSPBBR_3_QDS composite film will accumulate more VBR series and release in the core-shell interface of nanospheres, which is conducive to the formation of more nanoconductive channels. The memristor exhibits negative differential resistance as well as memory behavior, can endure more than 5,000 cycles, and remains stable for over 5 million seconds. Tong et al. employed quaternary Ag-In-Zn-S (AIZS) quantum dots to fabricate the cross-bar array.^120^ The power consumption can be as low as 10pW per switching. It is a potential contender for the development of energy-saving brain-inspired computing applications.

#### Material doping

**Figure 8:**
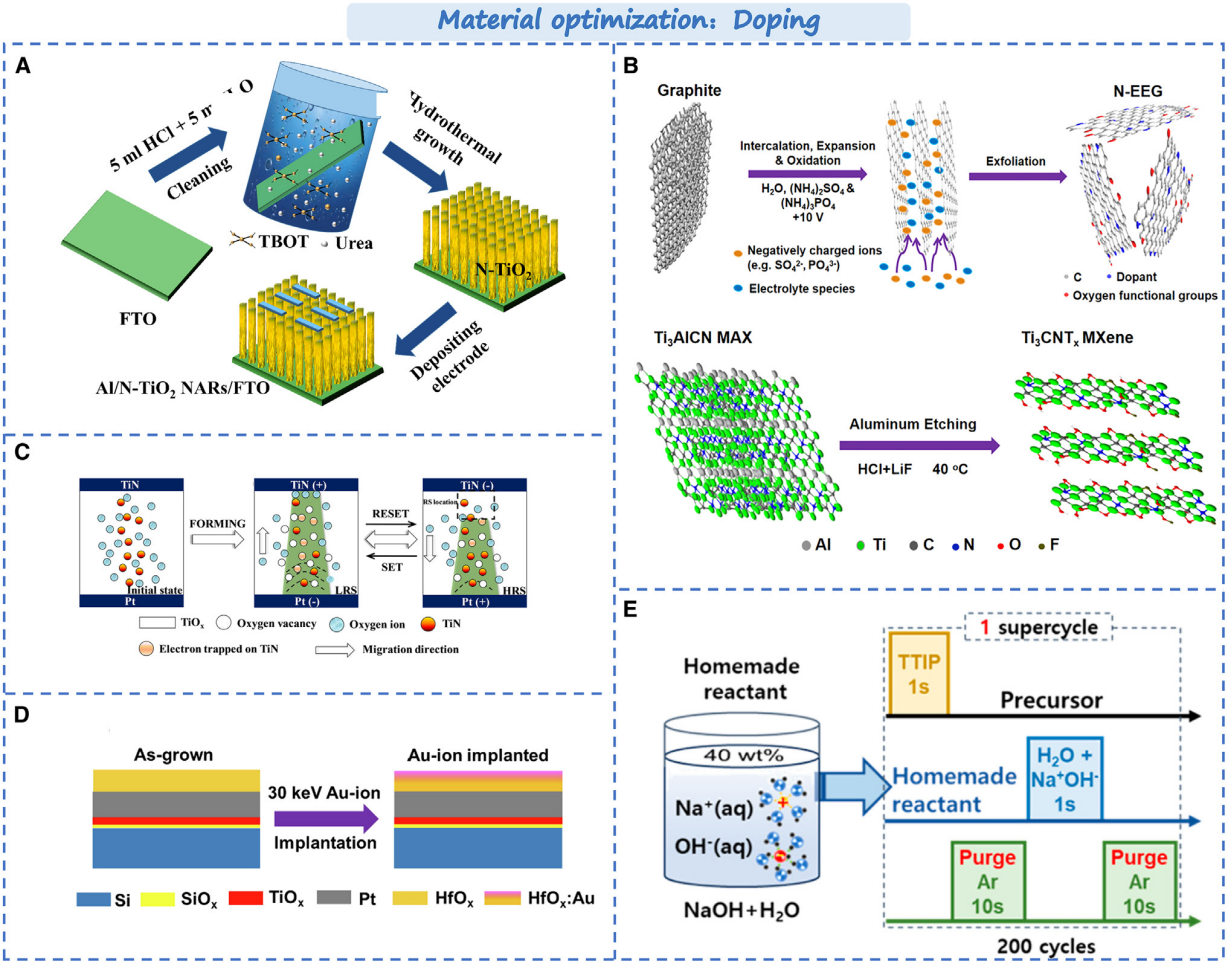
Optimization strategy based on memristor by material doping (A) The fabrication process of nitrogen-doped TiO_2_ nanorod array memristors.^123^ Copyright © 2021, Elsevier B.V. (B) The RS mechanism for the TiN/TiO_x_N_y_/Pt devices.^124^ Copyright © 2021, Wiley-VCH GmbH. (C) Synthesis process of N-EEG and Ti_3_CNT_x_ MXene.^29^ Copyright © 2024, American Chemical Society. (D) A schematic diagram of the devices under consideration.^125^ Copyright © 2022, Elsevier B.V. (E) Schematic representation of the Na/TiO_2_ ALD process.^126^ Copyright © 2024, American Chemical Society.

In addition to direct composition with materials, ion implantation (i.e., doping) is also one of the principal approaches to directly enhance the performance of devices. By doping certain elements into the material, the performance and function of the device can be improved, such as linearity, stability, and lifespan, etc.^121^^,^^122^ For the memristor, reducing the randomness of the conductive filaments and forming a stable and uniform resistance switch (RS) is the key to achieve neuromorphic computation. Wang et al. reported that doping nitrogen in TiO_2_ could enhance its resistance switching behavior.^123^ As shown in Figure 8A, through introducing nitrogen into the TiO_2_ nanorods array by hydrothermal synthesis, it provides more defects can further improve the conductance change process. The memristor exhibited excellent conductance continuous change ability and cycle stability. Similarly, Meng et al. reported the nitrogen-doped TiO_x_ memristor.^124^ As shown in Figure 8B, in based TiN/TiO_x_N_y_ devices, metal nanoparticles can promote the enhancement of electric field. When an electric field is applied, a uniform CF (i.e., an oxygen vacancy conducting filament) is readily formed near TiN electrode, which improved the RS performance. Additionally, as shown in Figure 8C, Dee et al. reported the nitrogen-doped graphene/Mxene memristors. The presence of the surface reaction site and the high conductivity stripping sheet can improve the performance of the memristor during operation. It can enhance the charge transfer, charge modulation, thermal conductivity, and stability of the device.^29^ Moreover, its temperature adaptability also can be improved. The feasibility of enhancing device performance through nitrogen element doping is further demonstrated.

In addition, the high linearity, symmetry, and stability of the resistance state in devices are of considerable significance for neuromorphic computation. Currently, it is challenging to enhance the linearity of most synaptic devices while guaranteeing the performance of the devices. However, the linearity and stability of the devices can be significantly improved through doping with metal ions. As shown in Figure 8D, highly stable gold ions are injected into HfO_X_. Owing to the presence of Au atoms near the top electrode. It can promote the formation of stable, non-random conductive filaments. The formation of specific structures or chemical states on its surface improves the transmission efficiency of electrical signals and the adjustment of synaptic weights.^125^ The linearity of synaptic weight enhancement can be notably improved. Furthermore, the device exhibits excellent RS characteristics. The essence of this characteristic improvement lies in achieving the modulation of the electrode interface schottky barrier or oxygen vacancy via the doping of metal ions. For example, Nahm et al. doped Cu^+^ into KNbO_3_/TiN films, leading to the increase of oxygen vacancies. This influenced the growth and dissolution of conductive filaments, ultimately enhancing the linearity of conductivity modulation.^127^ Lee et al. reported the Na^+^-doped Pt/TiO_2_/Pt memristor,^126^ as shown in Figure 8E. During the RS conversion process, Na^+^ doping at the lattice gap reduces the height of the Schottky barrier at the Pt/TiO_2_ interface, resulting in the occurrence of LRS, along with excellent stability and low volatility. At present, there have been numerous reports on the improvement of device performance through metal ion doping, such as Mn, Ag, Al, Lu, Ge, Cu, Fe, etc.^128^^,^^129^^,^^130^^,^^131^^,^^132^^,^^133^ The doping of metal ions will enhance the linearity and stability of the device and open up a new avenue for high-performance neuromorphic computing in the future.

#### Material modification and reconstruction

**Figure 9:**
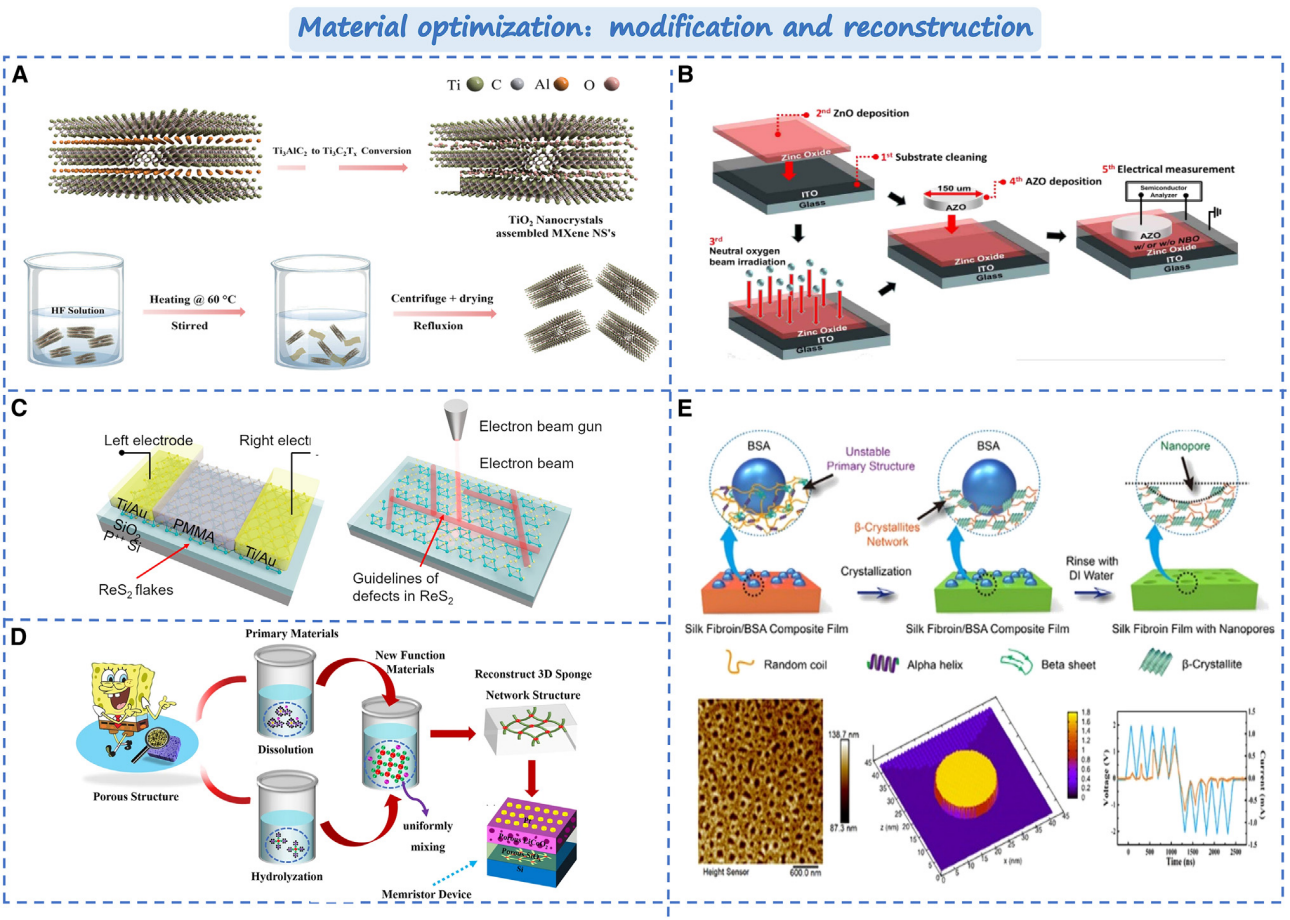
Optimization strategy based on memristor by material modification and reconstruction (A) The HF etching and reflux process of Ti_3_AlC_2_ to Ti_3_C_2_T_x_.^134^ Copyright © 2023, Wiley-VCH GmbH. (B) Schematic of device fabrication process flow.^135^ Copyright © 2022, Elsevier B.V. All rights reserved. (C) Structural of the the planar ReS_2_-based memristor.^30^ Copyright © 2021, Springer Nature. (D) The sponge-like double layer porous oxide memristor.^136^ Copyright © 2021, Springer Nature. (E) The p-SF film structure and fabrication process analysis.^137^ Copyright © 2021, American Chemical Society.

In the fabrication process of memristor material, the material is likely to lattice defects and grain boundaries. Moreover, the filament growth process is random, causing the device to exhibit random and unstable switching characteristics. Besides the approach of material composites, modifying the surface and morphology of the material can enhance the performance of the memristor. For example, operations such as thermal oxidation, annealing, and irradiation can improve the material defects. As shown in Figure 9A, Park et al. oxidized Ti_3_C_2_T_x_ to form TiO_2_ nanocrystals on the surface of the MXene layer and varied the conductivity of MXene (Ti_3_C_2_T_x_) by the oxidation degree.^134^ It can enhance the memory window of the device and reducing the threshold. Samukawa et al. employed neutral oxygen radiation to irradiate the surface of the based ZnO memristor,^135^ as shown in Figure 9B. Irradiation can effectively reduce the concentration of oxygen vacancy donor defects and promote the formation of oxygen gap acceptor defects on the surface of ZnO films, thereby regulating the filament breakage and regeneration process. Compared with the unirradiated device, the device’s lifetime is increased by 100 times, and the operating current can be reduced by 10 times. Similarly, as shown in Figure 9C, Ang et al. utilized an electron beam to irradiate the memristor of rhenium disulfide (ReS_2_) and further regulated the schottky barrier through the sulfur vacancy formed by irradiation, thus forming a stable gradient RS characteristic and comprehensively improving the device’s performance.^30^ It provides a simple and effective optimization strategy for high performance devices.

In addition, the bionic route design and natural porous structure within the memristor device can regulate the conductive channel and enhance the performance of the devices. Chu et al. proposed a category of spongy double-layer porous oxide memristor.^136^ As shown in Figure 9D, by emulating the hydrodynamic transport mechanism and water storage principle of water molecules in porous sponges, the switch ratio of the device the retention time can greatly improve. Moreover, nonlinear transmission characteristics, peak-time-dependent plasticity, and learning experience behavior can all be concurrently achieved through the device. Yan et al. also employed the protein surface reconstruction strategy to construct the porous fibroin (P-SF) memristor,^137^ as shown in Figure 9E. The reconstructed pores can direct improve the formation and fracture of Ag filaments, and enhance the conductivity by segregating the paths of Ag^+^ and electron diffusion. In this case, Ag^+^ can preferentially diffuse through the pores, while electrons diffuse through SF networks. Compared with non-porous devices, porous SF memristors have significantly enhanced electrical properties, including uniform I-V cycling. The optimization of the structure offers a convenient technical approach for high-performance material memristors.

## Optimization strategy based on memristor structure

In the practical application and research of memristors, device performance and stability are particularly important. However, the performance of the memristor still faces numerous challenges, including device consistency, resistance uniformity, and leakage current generation in memristor arrays.^138^ Comparing the optimization from material aspects, structural alterations offer increased flexibility and scalability. In this section, some improvements of memristors based on memristor structures are listed. Such as: electrode engineering, heterojunction, etc. Then some new structures based on memristors are listed.

### Design and optimization strategy in electrode structure

**Figure 10:**
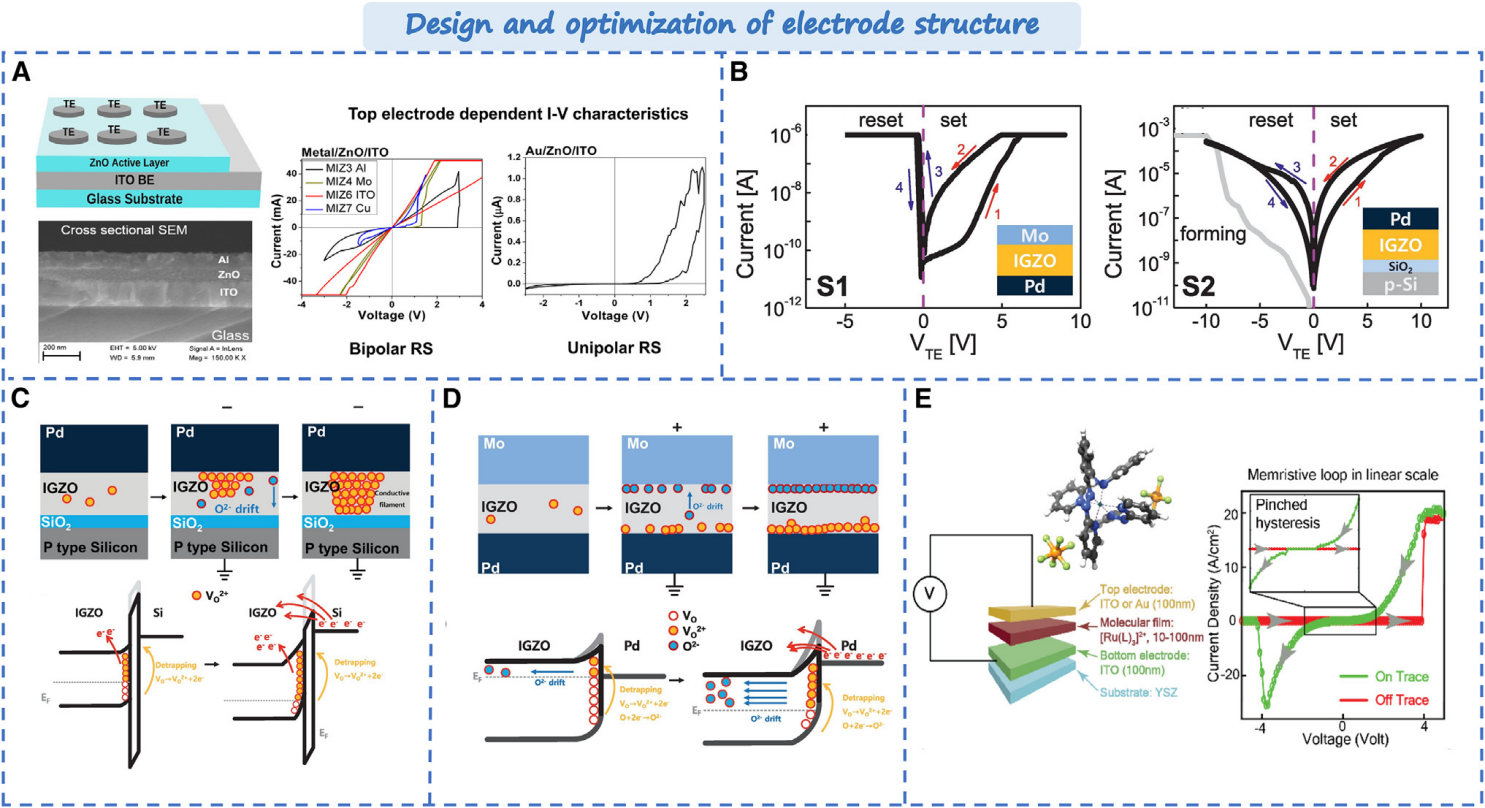
Design and optimization strategy in electrode structure (A) Schematic of the ZnO/ITO based memristor device structure and I-V characteristics.^31^ Copyright © 2022, Elsevier. (B) Memristor characteristics of two kinds of device models.^139^ (C) Switching mechanism of Mo/IGZO/Pd device. (D) Switching mechanism of Mo/IGZO/SiO_2_/Pd device. © 2022, Elsevier Ltd. (E) The device structure and I-V characteristics.^140^ Copyright © 2020, Elsevier Ltd.

The electrode material of the memristor not only serves as the conductor, but also involves oxidation-reduction reactions in specific structures, participating in resistive switching processes. The selection of electrode materials determines the conductivity and stability of the device. The electrode materials mainly include metal materials, carbon-based materials (graphene and carbon nanotubes), nitrides (TiN and TaN), transparent conductive flexible oxides (ITO, FTO), etc. The electrode structure can be symmetrical or asymmetrical. When the electrode material is the active electrode material (such as Ag), it directly affects the conduction type of the memristor. For example, Saji et al. achieved different conduction mechanisms by using Al, Cu, Au as top electrodes.^31^ Valence change and electrochemical mechanisms can be observed in filamentary resistive switching. As shown in Figure 10A, Al, ITO, and Cu top electrodes exhibited bipolar and filamentary resistive switching behavior while the unipolar interface resistive switching was achieved in Au/ZnO/ITO devices. Similarly, as shown in Figure 10B, Kim reported that it can modulate Schottky barrier heights at metal-semiconductor interfaces by selecting two different types of electrode materials.^139^ In Mo/IGZO/Pd devices, under positive biasing conditions, electrons near IGZO/Pd interface Schottky barrier are released when VO^2+^ are induced due to positive bias. As shown in Figure 10C, it leads to modulation of Schottky barrier height resulting in resistance decrease during set process. Conversely under negative biasing conditions, O^2+^ drifts neutralizes vacancies leading to modulation back to original height during reset process. In Pd/IGZO/SiO_2_/P-Si devices, thermal electron emission is the main conduction mechanism. When oxygen vacancies are ionized under positive bias, it will lead to the increase in VO^2+^. As shown in Figure 10D, it can adjust the potential barrier height of the SiO_2_ layer causing the decrease in resistance. Furthermore, the “electrode engineering” has been proposed by improving interfaces and shapes of electrodes to enhance metal-semiconductor contacts. As shown in Figure 10E, Venkatesan et al. successfully improved contact between electrodes by manipulating characteristics and geometrical shape, achieving significant adjustability (approximately 2500% variation) in switch voltage (from 130mV to 4V) and current (approximately 6 orders).^140^ Ahn et al. reported the Nb/NiO/Nb memristors with well-aligned bottom electrodes consisting of a Nb nanoneedle array.^141^ The enhanced induced electric fields led to lower SET/RESET voltages. The minimized dispersion in LRS/HRS currents and SET/RESET voltage. It further highlights the significance of design in improving device performance from both structural and material perspectives.

### Optimization strategy in the RS layer

**Figure 11:**
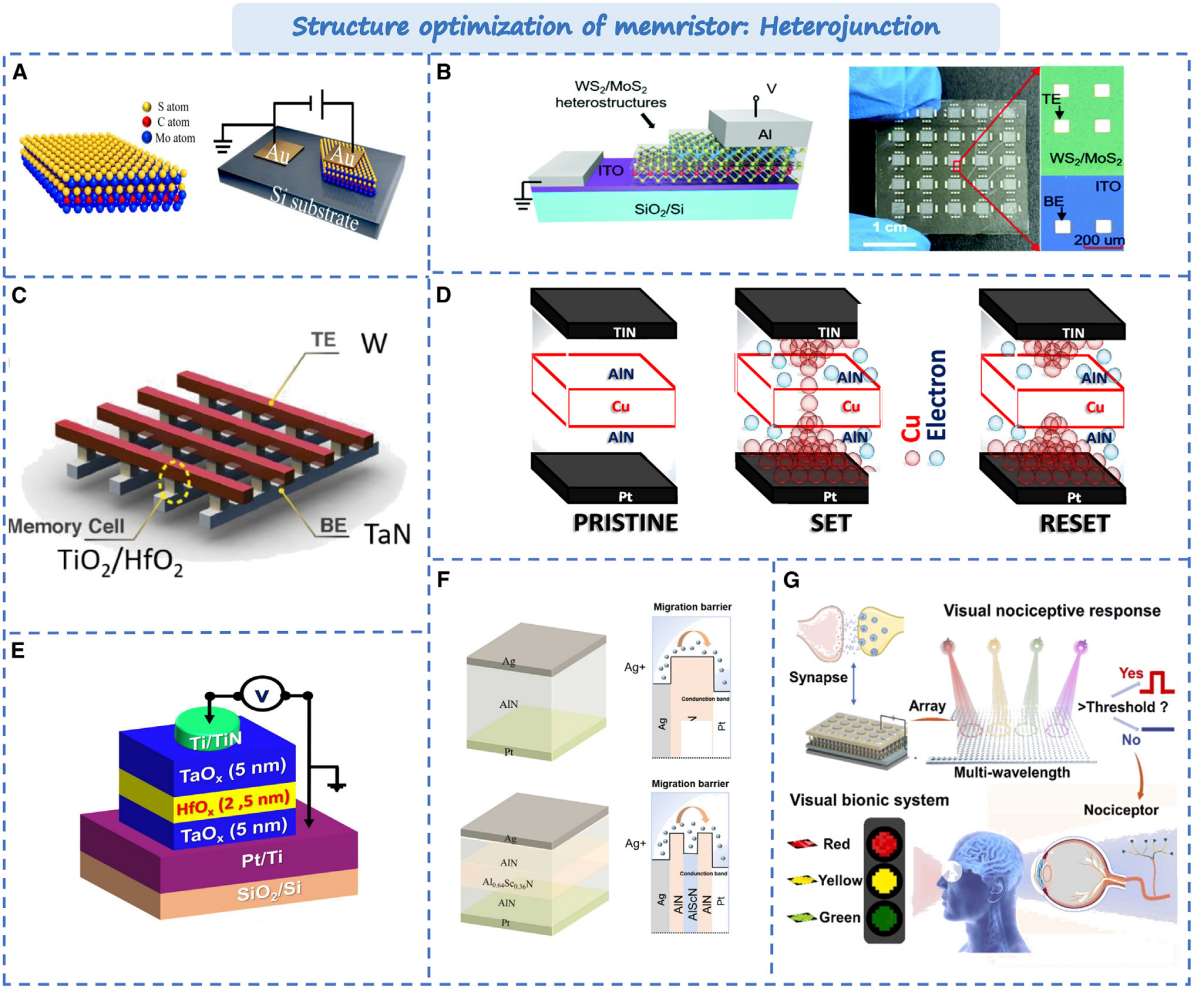
Typical optimization strategy in the RS layer (A) Schematic illustration of the Mo_2_C/MoS_2_ heterostructure.^144^ Copyright © 2022, Springer Nature. (B) The WS_2_/MoS_2_ heterojunction memristor.^32^ Copyright © 2021, Royal Society of Chemistry. (C) The heterostructure-based crossbar array.^145^ Copyright © 2022, Elsevier. (D) Conductive filament model of the TiN/AlN/Cu/AlN/Pt device in the set/reset states.^146^ Copyright © 2023, American Chemical Society. (E) The TaO_x_/HfO_x_ Bi-layer memristor.^147^ Copyright © 2022, Wiley-VCH GmbH. (F) The band diagram of the RS mechanism for Ag/AlN/Pt memristor.^148^ Copyright © 2024, Elsevier Ltd. (G) An pain-sensing artificial visual system.^149^ Copyright © 2024, Elsevier Ltd.

With the emergence of new materials, such as two-dimensional materials, metal oxides, organic polymers, perovskites and so on, heterojunction technology plays a crucial role in providing material diversity and functional uniqueness.^142^ By stacking various materials, adjusting the band structure, and controlling electron transport, the devices performance can get improved.^143^ In based on two-dimensional materials memristors, the durability and stability of the device are poor due to the thinness of two-dimensional materials. To solve this problem, forming VdW homogeneity or heterojunction by stacking two-dimensional materials can further enhance device performance. Gui et al. reported a category of homologous Mo_2_C/MoS_2_ memristors.^144^ The device structure is shown in Figure 11A. Its performance got greatly improved comparing the single layer, including a retention time of up to 10^4^s, durability of up to 100 cycles, and an on/off ratio of up to 10^3^. Moreover, the built-in electric field of the P-N junction has a direct impact on the resistance state of the memristor. During the operation of the memristor, modulation of the P-N junction can influence the injection and extraction of charge carriers, thereby altering the conductivity state of the memristor. Xiong et al. proposed a novel memristor structure based on the WS_2_/MoS_2_ heterojunction as shown in Figure 11B.^32^ Due to the differences in the energy band structures of materials, the formed bidirectional potential barriers exhibit a significant blocking effect on electron transport. By modulating the energy band structure of heterojunctions through an electric field, reversible resistance switching can be achieved. Compared to conductive filaments, the resistance switching characteristics based on energy band structure modulation demonstrate higher stability and excellent repeatability. It’s switching ratio ups to 10^4^ and overs 120 switching cycles, demonstrating that the 2D WS_2_/MoS_2_ heterojunction is superior to the single MoS_2_ or WS_2_ layer in memory performance.

Furthermore, by precisely controlling the composition and thickness of the heterostructure, the resistive switching characteristics of the memristor can be regulated, enabling more refined current control and lower power consumption. For example, by inserting 2 nm BiFeO_3_ in the HfO_2_-based memristor, the device can achieve a 10^4^ storage window and a 10^8^ s retention time.^145^ As shown in Figure 11C, Jin et al. used TiO_2_/HfO_2_ memristor as the memory unit, and the gradual switching characteristics of the bilayer memristor device had a lower operating voltage and better switching uniformity.^150^ Therefore, by inserting oxide layers in single-layer memristors and controlling the distribution of oxygen vacancies, the electrical performance of devices can be improved. Currently, there have been numerous reports based on oxides heterostructures, such as NiO_x_/WO_3−x_:Ti, TaO_x_/ITO, TiO_2_/HfO_2_, Ta_2_O_5_/HfO_2_, ITO/Ta_2_O_5_, IGZO/HfO_2_, V_2_C/TiO_2_, HfO_x_/SnO_x_, IGZO/ZnO, etc.^151^^,^^152^^,^^153^^,^^154^^,^^155^^,^^156^^,^^157^^,^^158^ Additionally, to better control the formation process of conductive filaments, using the three-layer structure can more stably control the formation of conductive filaments, resulting more uniform switching characteristics. As shown in Figure 11D, by inserting Cu into amorphous AlN layers, it forms conductive filaments based on the oxidation and dissolution of Cu ions. The interface-type switch can generate better progressive conductance modulation characteristics than the filamentary switch.^146^ As shown in Figure 11E, when HfO_x_ is inserted between the TaO_x_ layers, the devices possess more stable switching behavior low set/reset voltages and multi-level cell characteristics.^147^ From the perspective of energy bands, the three-layer structure is more inclined to form a quantum well structure. As shown in Figure 11F, The resulting potential well and ferroelectric polarization effect hinder the migration of electrons and ions in the AlN layer, which can avoid the sudden formation/fracture of conductive filament (CF).^148^ The conductance state is gradually changed and the linearity of conductance is optimized. This provides a new structural paradigm for achieving more efficient and accurate neuromorphic computing.

In addition, the based heterostructure memristor shows more stable working state in extreme environments, such as high temperature, folding, bending, and other application scenarios. For example, Park et al. reported the HfO_x_/AlO_y_ memristor, which enhanced thermal stability in a wide working temperature range of 25°C–145°C.^159^ Similarly, Tseng et al. reported the Ta_2_O_5_/WO_3_ and TaO_x_/HfO_x_ memristors can keep stable performance under extreme weather conditions.^160^ This provides a novel possibilities for their application in special environments. Furthermore, memristors based on heterogeneous structures offer new opportunities for the new functional development. For example, as shown in Figure 11G, Yang et al. realized the perception, memory and color recognition of “traffic signal” image human vision system based on CeO_2_/MoS_2_ photoelectric memristor multi-wavelength response to photon signal.^149^ It presents a strategy for the integration of sensing, memory, and visual pain devices for electronic eyes and humanoid robots in the future. Furthermore, memristors based on heterojunctions have been utilized in the fields of touch, smell, hearing, and touch. It proposes more possibilities for the future application of memristors.

### Emerging devices based on memristors

**Figure 12:**
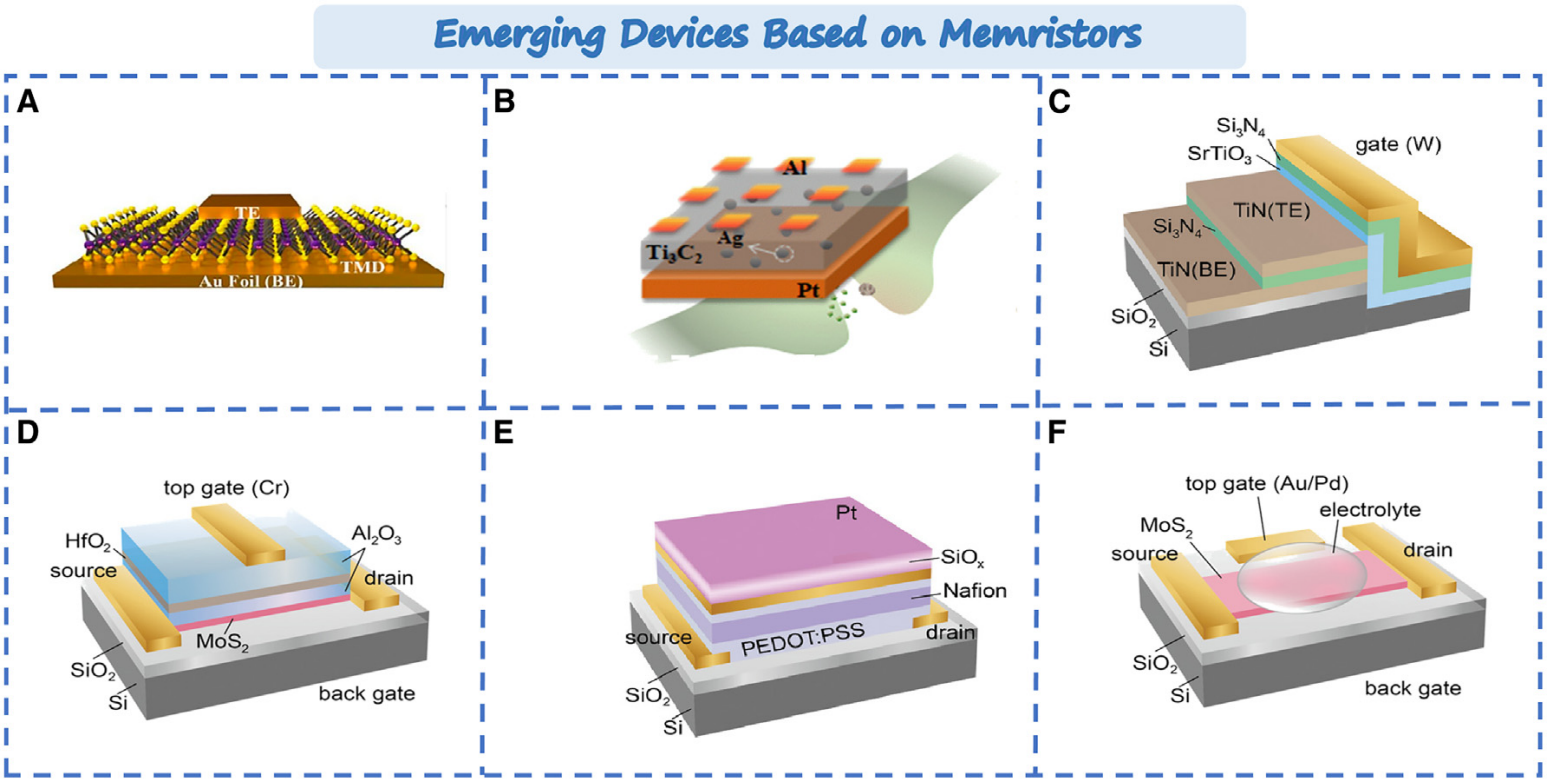
Emerging devices based on memristors (A) Vertical structure memristor.^161^ Copyright © 2017, American Chemical Society. (B) Multi-input vertical structure memristor.^162^ Copyright © 2020, Elsevier Ltd. (C) Three-terminal side-gate memtransistor. (D) Dual-gated MoS_2_ charge trapping memory device. (E) Floating-gate memory device. (F) Planar dual-gate memristor.^164^ Copyright © 2022, Wiley-VCH GmbH.

Currently, the structure of memristors mainly includes vertical and planar structure. Vertical memristors can control the distance of the RS layer reaching the nanometer or sub-nanometer level. It can low switching voltage of memristors. For example, 2D materials can achieve nanoscale thickness, and this structure can well utilize the advantages of 2D materials. As shown in Figure 12A, Akinwande et al. reported the vertical memristors based on 2D materials (such as MoS_2_, WS_2_, MoSe_2_, and WSe_2_).^161^ The devices exhibited bipolar resistive switching behavior with an on/off ratio exceeding 1×10^4^. Furthermore, vertical memristors provide the great advantage in three-dimensional integration of neuromorphic devices. Besides, in order to better show the processing capability of multiple information, multi-input vertical memristors have been proposed.^162^ As shown in the Figure 12B, multiple top electrodes are added on the upper surface of the dielectric layer. When signals reach to the top electrode, multiple signals can be integrated in the same RS layer. Furthermore, planar memristors can achieve greater breakthroughs in structure. For example, in 2018 Hersam et al. proposed the concept of “memtransistor”.^163^ The memtransistor combines the concepts of memristor and transistor. It provides an additional control terminal for the memristor. The gate terminal not only modulates the transmission characteristics of the transistor, but also modulates the RS behavior of the memristor. It brights new possibilities in multi-information storage. At the same time, various structures of memtransistor have been explored.^164^ As shown in Figure 12C, the lateral insulated-gate memtransistor can modulate the resistance between the first two terminals by 20 times. As shown in Figure 12D, dual-gated MoS_2_ charge-trap memtransistors can achieve low-power computation. Figures 12E and 12F, respectively, illustrate double-gate memtransistors based on floating gate structure and planar structure. Through multi-gate control, better control over resistive states can be achieved. These provide a solid foundation for integrating high-performance neuromorphic devices.

## Optimization strategy based on memristor array

**Figure 13:**
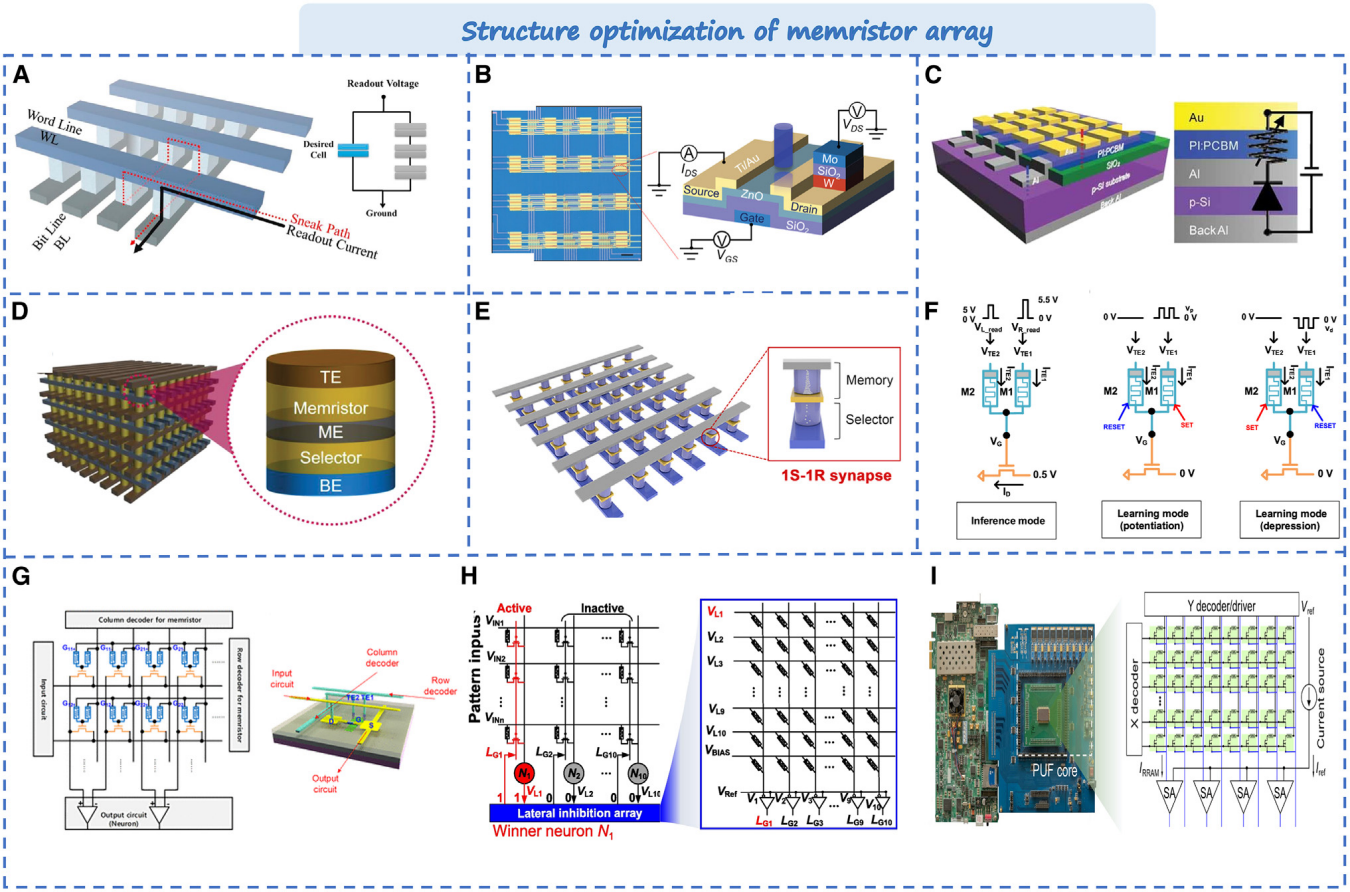
Typical optimization strategy based on memristor array (A) The sneak-pathcurrent in crossbar memory device.^33^ Copyright © 2019, Elsevier. (B) The structure of 1PT1R memristor array.^165^ Copyright © 2022, Wiley-VCH GmbH. (C) The 1D–1R hybrid-type memory devices.^166^ Copyright © 2010, WILEY-VCH Verlag GmbH & Co. KGaA, Weinheim. (D) 3D integration of 1S1R crossbar arrays.^167^ Copyright © 2019, WILEY-VCH Verlag GmbH & Co. KGaA, Weinheim. (E) The neural network based on the electrochemical metallization memristors.^34^ Copyright © 2019,Wiley-VCH GmbH. (F) The principle of the 1T2M synaptic device. (G) The neuromorphic system of 1T2R synaptic array.^168^ Copyright © 2020, American Chemical Society. (H) Schematic of the lateral inhibition array circuit.^169^ Copyright © 2021, Elsevier B.V. and Science China Press. (I) Photograph of the PUF system containing an 8-kb memristor array.^170^ Copyright © 2022, The American Association for the Advancement of Science.

To achieve higher memory density in computers, energy efficiency is an important factor in shaping the future of information technology. The based memristor crossbar array is anticipated to replace the traditional computing architecture due to its high memory density and low energy consumption. This presents significant potential for application in future computing architecture. However, in the actual crossbar array, expecting the performance and stability of the memristor, as shown in Figure 13A, stealth current caused by adjacent elements is a difficulty in the three-dimensional integration of crossbars.^33^ Currently, the solution for sneak path issues involves dynamically controlling their paths during operation. This approach ensures that specific paths are only activated when necessary, thereby guaranteeing accurate signal transmission and processing. In this section, we primarily focus on discussing the methods used to resolve sneak path problems in device arrays.

Currently, the primary approach involves connecting basic electronic components with each memristor unit, such as diodes, resistors, selectors, transistors, and so on. This method can prevent interference and suppress leakage currents. For example, Huang et al. connected phototransistors with each memristors, as shown in Figure 13B.^165^ The 1PT1R devices demonstrated highly linear weight updates based on optical programming and highly uniform multistage conductivity states in crossbar array. Another approach to reduce latent path current is connecting a double-ended selector to each unit. Li et al. developed a hybrid device composed of a Schottky diode and a memristor (1D-1R), as shown in Figure 13C.^166^ By utilizing the superior rectifier characteristics of the diode, it can regulate the resistance state of the memristor by controlling the voltage. The 1D-1R device is simpler in design and manufacture compared to the 1T-1R device, reducing the complexity for high-density integration processes. Additionally, Pan et al. proposed a novel integrated 1S1R (selector and memristor) based on three-layers oxides, as shown in Figure 13D.^167^ The homojunction selector exhibits high insulation and low ion mobility, allowing for the modulation of silver conductive wire migration and corresponding cations. This capability effectively prevents unnecessary cross-talk current. In order to address voltage matching issues in the construction of a 1S-1R device with high integral density, Li et al. conducted optimization of electrical properties in electro chemical machining (ECM) devices by dispersing active metal nanoparticles at the interface, as shown in Figure 13E.^34^ Compared to previously reported devices, this device exhibits a high on/off ratio, excellent selectivity, low operating current, and stable multilevel conductance. Its neural network of this device array demonstrates reliable parallel computing capability and high energy efficiency.

In addition to single-device connections, Kim et al. proposed a category of single-transistor-dual memristor (1T2M) structures, as shown in Figure 13F.^168^ The state of the transistor can be controlled by manipulating the positive or negative voltage input to the memristor. The 1T2M synaptic array is shown in Figure 13G. The 1T2M synaptic device demonstrated more linear and symmetrical conductance modulation characteristics compared to single-device connections. Additionally, this array structure showed good robustness against the stealth path problems. Liu et al. combined a memristor with a simple digital circuit to design a hybrid pulse neuron, as shown in Figure 13H.^169^ It marks the first experimental realization of an all-hardware pulse neural network with hybrid neurons and memristor synapses. Furthermore, Wu et al. employed complementary metal oxide semiconductor (CMOS) technology to design a more compact memristor array-based hidden PUF.^170^ As shown in Figure 13I, the hafnium oxide memristor can effectively hide/recover PUF through SET/RESET operation. PUF recovery boasted a zero bit error rate and outstanding anti-attack capabilities with negligible power consumption. This offers a new promise for the development of more secure memristor hardware systems in the future.

## The application of memristors

Due to the unique physical characteristics of memristor, such as memory, nonlinearity, low power consumption, stability, and ease of constructing cross arrays, it is considered the ideal component for simulating the human brain neural network. With the increasing focus on research into the material, structure, and mechanism of memristors, it has shown significant potential for various applications, including conventional memory storage techniques, neuromorphic computing, and multi-modal perception.^171^^,^^172^ This section will primarily discuss the current applications of memristors.

### Multi-modal perception

With the rapid advancement of science and technology, the world is undergoing swift changes, and a lot of external information is emerging. Therefore, it is of great significance for artificial intelligence to precisely receive and process external information. Memristor, as a novel type of artificial synaptic device, naturally possesses superiority in simulating human senses. By combining memristors with sensors, it becomes possible to simulate human sensory perceptions such as hearing, smell, vision, touch, etc., thereby achieving the perception of external information and interaction.

**Figure 14:**
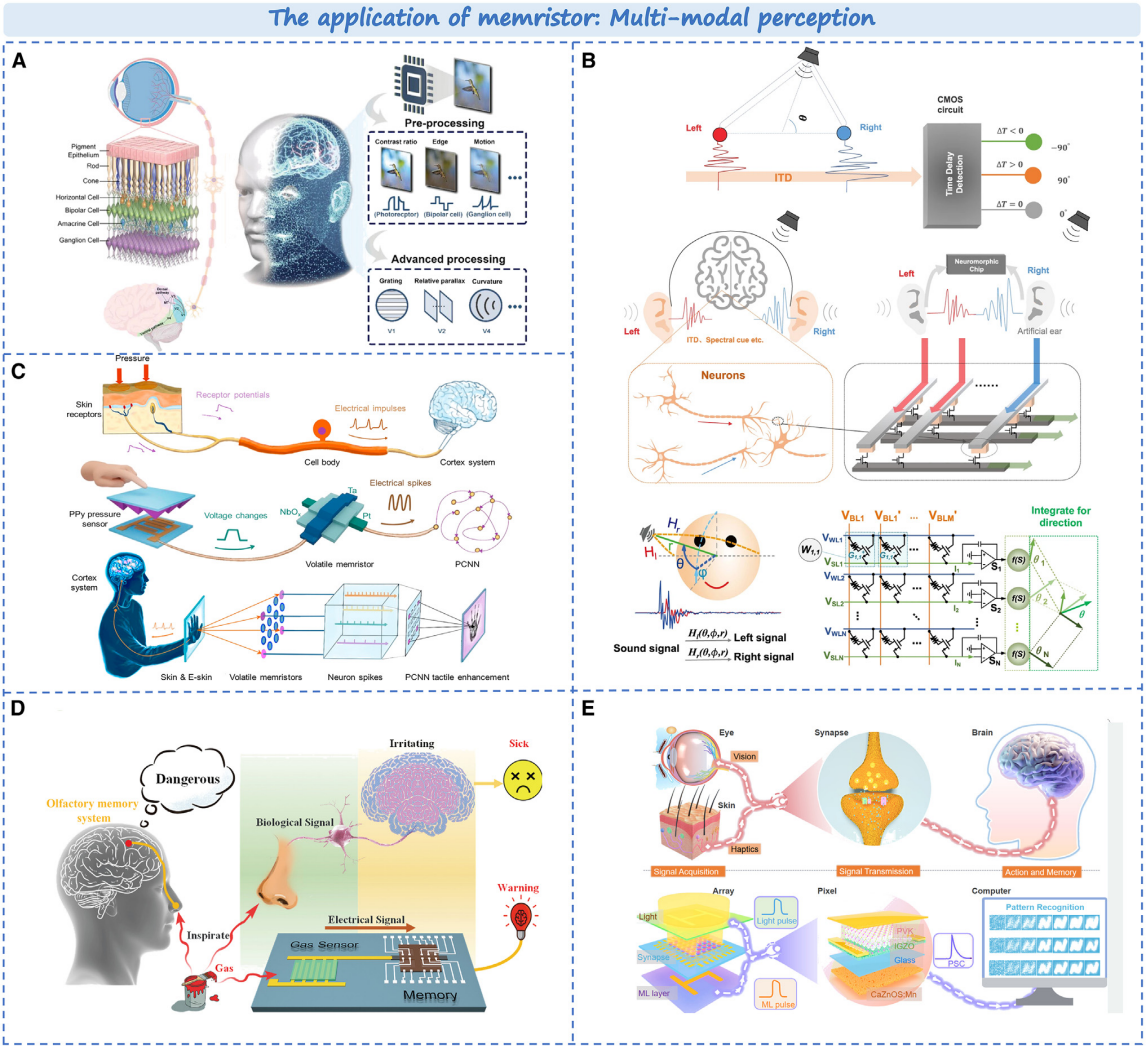
Typical application of memristors in multi-modal perception (A) The biological visual pathway and the artificial visual processing system.^177^ Copyright © 2023, Wiley-VCH GmbH. (B) The sound localization and hardware implementation.^180^ Copyright © 2022, Springer Nature. (C) Biological and artificial mechanoreceptors.^181^ Copyright © 2021, American Chemical Society. (D) The artificial olfactory memory system.^182^ Copyright © 2021, Wiley-VCH GmbH. (E) The integration of human visual-tactile perception system and artificial visual-tactile perception array.^35^ Copyright © 2024, UESTC and John Wiley & Sons Australia.

In traditional machine vision, the vision system typically consists of image sensors, digital-to-analog converters and storage units. The computing units are usually implemented using traditional CMOS technology,^173^ which results in poor speed and power consumption during data transmission between memory and computing units. In contrast, the human visual system is a highly integrated system for perception, storage, and computing.^174^^,^^175^^,^^176^ By emulating the unique structure and mechanism of the natural eyes. As shown in Figure 14A, bionic retina can be imitated by neuromorphic devices and circuits to realize more energy-efficient and biological artificial visual hardware.^177^^,^^178^^,^^179^ This effectively enables the realization of visual cortex functions such as image recognition and classification, making it a potential candidate for constructing new visual systems. Furthermore, sound source detection (i.e., hearing) is a fundamental function of human beings. Wu et al. proposed a brain-like algorithm and architecture based on memristor. As shown in Figure 14B, an array integrated with 1K memristor units can process the complete sound signal received by two artificial ears.^180^ Its power consumption is reduced by approximately 184 times compared to existing ASIC designs while maintaining processing accuracy. This paves the way for building neuromorphic hearing systems.

When human skins contact with the surrounding environment, it can sense objects through touching. Memristors, with simple double-ended structure and dynamic threshold switching, are highly suitable for the artificial tactus. By integrating memristor units into artificial tactile systems, it is feasible to enhance the sense by artificial machines. In Figure 14C, Yue et al. proposed a type of artificial mechanoreceptors consisting of a micropyramid-resistance pressure sensor and a volatile memristor.^181^ It can complete the perception, processing and storage of external information. This simulation of tactile perception and processing lays the groundwork for future applications, such as controlling humanoid robots and prosthetics. Similarly, the integration of gas sensors and memristors can be used to simulate the artificial olfactory memory system. As shown in Figure 14D, the system exhibits exceptional selectivity for volatile organic compounds.^182^ When the gas concentration surpasses a certain threshold, it retains olfactory information, leading to gas recognition. This intelligent olfactory memory system has direct applications in various robotics and artificial intelligence systems, including environmental pollution control, early warning systems for chemical and biological hazards safety, as well as enhanced intelligent industrial production.

Finally, the ultimate goal of developing humanoid robots and cross-modal human-machine is to replicate the multi-sensory function of humans to establish a complete artificial perception system. Inspired by human multisensory signal generation and neuroplasticity-based signal processing, Pan et al. demonstrated an artificial perceptual neural array with vision and tactile sensing, processing, learning, and memory.^35^ As shown in Figure 14E, The neuromorphic bimodal sensing array closely integrates an artificial photoelectric synaptic network with integrated mechanoluminescence layer. This allows for individual and collaborative plastic modulation of optical and mechanical information, including short-term memory, long-term memory, paired pulse promotion, and learning experience behavior. Continuous or superimposed visual and tactile stimulus inputs can effectively simulate the associative learning process witnessed in Pavlov’s dog. Owing to its mechanical compliance and simple architecture, neuromorphic bimodal sensing arrays have extensive application prospects in large-scale cross-modal interaction and high-throughput intelligent sensing. This constitutes the first step on the path toward future multi-modal integration.

Currently, AI systems based on memristors mainly involve tactile and visual functions, which are rare in other human brain-like functions. Memristor-based neuromorphic chips, as typical representatives in AI applications, can promote the hardware implementation and terminalization of AI systems. In addition to simulating the senses of AI, memristors also have significant implications for other aspects of artificial intelligence such as cloud computing, autonomous driving, neuromorphic chips, and big data processing. However, the construction of AI hardware systems currently only remains at a single function level. Achieving an AI system with high speed, low power consumption, high density, small size, good versatility, low cost and even environmental friendliness is still a considerable distance away. The continuous challenge lies in how to integrate memristor-based systems with multiple functions similar to those found in the human brain. They face various challenges related to devices, arrays/macroscale structures and algorithms. Future efforts should focus more on practical applications in the future.

### Memory storage techniques

**Figure 15:**
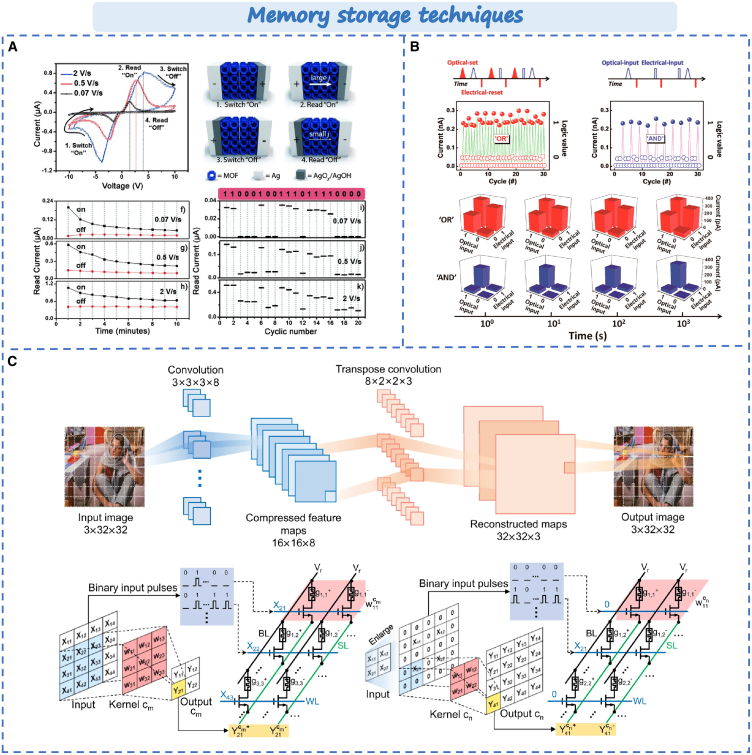
Typical application of memristors in memory storage techniques (A) An electrically addressable MOF memory illustrating reading, writing, and erasing capabilities.^183^ Copyright © 2014, WILEY-VCH Verlag GmbH & Co. KGaA, Weinheim. (B) Schematic diagram of data storage and logical calculation.^184^ Copyright © 2014, American Chemical Society. (C) Schematic illustration of the proposed near-storage in-memory processing system implemented with memristor-based cores.^185^ Copyright © 2014, Springer Nature.

In recent years, memristors have become strong competitors in information storage technology owing to their outstanding non-volatility, high storage density, low power consumption, durability and multi-value storage capability. For example, Grzybowski et al. achieved electrical information storage using single metal-organic frameworks (MOFs) crystals.^183^ As shown in Figure 15A, the sub-nanometer width channels in MOFs only allow small ions to pass through. In contrast, the presence of an electrolyte without MOF does not exhibit memristance or memory effect. This enables the persistence of resistive states even without applied voltage. By altering the polarity of voltage, electrode oxidation-reduction states can be manipulated to achieve write-in states ("1" or "0"). In addition, this type of memristor boasts a data retention period lasting up to one week. Additionally, wet stamping and other techniques can be used to achieve high-density data storage in MOF-based memristors by patterning multiple electrodes on MOFs. In addition to achieving information storage through electrical signals, Li et al. integrated non-volatile memory into a single optoelectronic gate-type memristor based on its non-volatility and sustained photoconductive effect.^184^ As shown in Figure 15B, the possibility of attaching memory functions to logic units can eliminate additional RAM blocks in traditional programmable logic circuits. It can help address data transfer bottlenecks in current state-of-the-art von Neumann architecture computer systems. Furthermore, Kang et al. proposed a memristor-based storage system.^185^ As shown in Figure 15C, it shows the integration of convolutional autoencoder compression networks based on near-storage memory computing. This integration aims to improve energy efficiency and speed for image compression/retrieval, while also increasing storage density compared with server-level central processing unit/graphical processing unit-based processing systems. The delay and energy consumption are reduced by 20×/5.6× and 180×/91× respectively, and the storage density is increased by over 3 times. This brings new possibilities for future high-density storage systems.

### Neuromorphic computing

**Figure 16:**
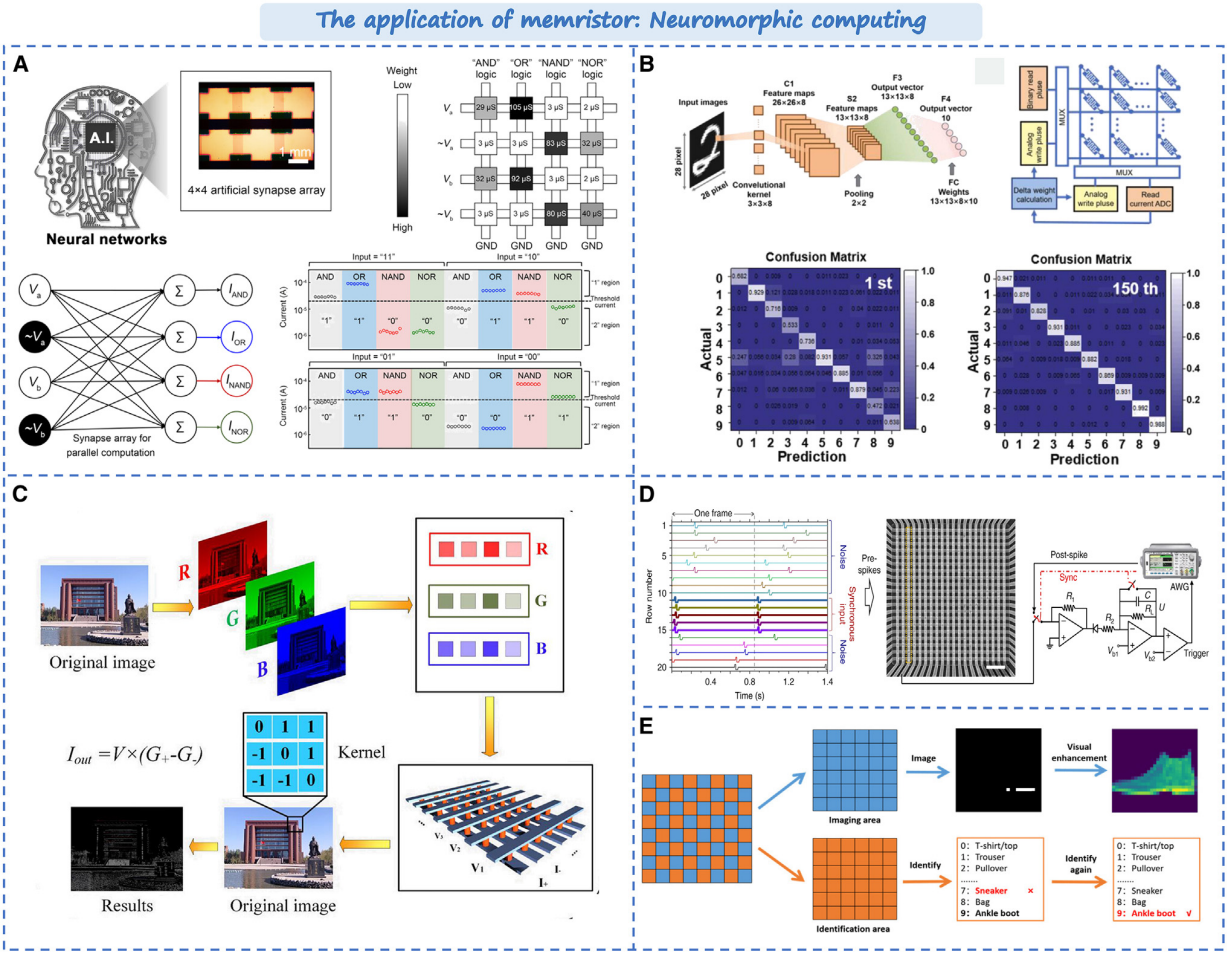
Typical application of memristors in neuromorphic computing (A) Logic operation of the memristor-based neural networks.^34^ Copyright © 2022, Wiley-VCH GmbH. (B) The simulated convolutional neural network (CNN) structure.^159^ Copyright © 2024 Elsevier Ltd. (C) Processed image results using memristor array.^186^ Copyright © 2023, American Chemical Society. (D) The hardware system of the crossbar circuit.^187^ Copyright © 2018, Springer Nature. (E) The vision-enhanced in-sensor computing neural network.^188^ Copyright © 2018, Multidisciplinary Digital Publishing Institute.

Neuromorphic computing, also known as brain-like computing or neuromorphic engineering, is a computational approach inspired by the human brain. Its aim is to emulate the information processing patterns and structures of biological nervous systems. Differing from the traditional computing paradigm, it is based on pulse neurons to realize efficient information processing with low power consumption and low delay. In the diverse applications of neuromorphic computing systems, such as artificial intelligence and pattern recognition systems, it is essential to simulate vector-matrix multiplication in synaptic arrays. Lee et al. developed a 4 × 4 1S-1R synaptic array.^34^ As shown in Figure 16A, it compolished Boolean logic operations. Additionally, the average computational energy per logic operation is approximately 1.28 pJ, demonstrating its feasibility in futural energy-efficient neural networks. Moreover, memristors can utilize synaptic weights to mimic losing function in neural networks in the training of neural networks. Hu et al. accomplished handwritten digit recognition using synaptic resistance weights.^148^ As shown in Figure 16B, the neural network model attained a recognition rate of 93% when utilizing the curve fitted by device conductance, demonstrating its potential for neuromorphic computation. Furthermore, Yan et al. converted the pixel values of the color image to the voltage signal, and then inputed to the STO:MgO memristor array, as shown in Figure 16C.^186^ The convolution operation can speed up the processing of information and significantly reduce power consumption in traditional big data computing applications, such as automatic driving, brain-computer interfaces, and pattern recognition.

Merely employing the parameters of the device to achieve neuromorphic computing is insufficient to solve the architecture constraints. Constructing the hardware neural network model using synaptic devices is essential for brain-like computation. Strukov et al. developed a hardware pulse neural network (SNN) utilizing memristors, as shown in Figure 16D.^187^ The SNN allows it to perform coincidence detection tasks based on software pulse neural networks. It establishes a solid foundation for constructing hardware-based neural network models. In addition, fully optical memristors have unique advantages in neuromorphic computing, such as high bandwidth, good robustness, and low energy consumption. It shows great potential in building the next generation of artificial intelligence and neuromorphic computing systems. Currently, there have been numerous reports on fully optical memristors.^90^^,^^189^ Many synaptic functions such as LTP, STDP, etc. can be achieved through optical signals. What is more, Chen et al. reported that the retinomorphic memristor can achieve stable image processing based on all-optical light control.^190^ The recognition rate of processed images reaches 83.5% after 19,000 iterations, surpassing the performance of fuzzy images (which only reach 56.2% after 19,000 iterations). It shows good anti-noise ability. As shown in Figure 16E, Liu et al. proposed an AI vision scheme capable of simultaneously imaging and recognizing, while dynamically adapting to environmental conditions.^188^^,^^191^ In low-light conditions, the readout voltage can be adjusted to increase light sensitivity and enhance the quality of the captured images. This approach allows for non-volatile storage of weight values and reduces average power consumption across the entire sensor chip compared to adjusting optical responsiveness via readout voltage. Similarly, in low-light environments, the readout voltage can be synchronously adjusted with the imaging unit to enhance image recognition accuracy. This enables dynamic adjustments in imaging, recognition, and calculation results based on environmental light intensity within the computing chip embedded in the sensor.^192^ It opens a new chapter for the construction of neuromorphic hardware systems in future.

## Summary and outlook

The memristor optimization is a comprehensive process, including material selection, device fabrication, and structure design. During the device fabrication stage, it is important to consider various factors. This paper provides a review of commonly used process techniques, such as CVD, ALD, sputtering, and 2D/3D printing, and analyzes their influence on device performance. Additionally, recent improvement strategies for materials are discussed including materials composite and modification. Furthermore, the impact of heterojunction structure on device performance is investigated from an energy level perspective. The paper also discusses leakage current in cross arrays. Finally, the latest applications of memristors in neuromorphic computing and multimodal sensing are introduced. Despite remarkable achievements in recent years regarding the performance and application of memristors, several challenges and limitations still exist. (1) For device performance, it depends to a large extent on the material used. However, the further exploration of good electrical properties in such electronic material is still a challenge. Moreover, the consistency, stability, and reliability make a great trouble of the memristors from the practical applications. (2) Secondly, while there is potential for improved performance through material and structural enhancements, they would also lead to the increasing complexity and cost in the production process. Furthermore, there remains a significant gap between current capabilities and actual production. (3) In a cross array of memristors, inherent defects arise due to the working principle of memristor. These defects include device fluctuations, conductance lag, and conductivity state drift, which ultimately results in a reduced calculation accuracy. Additionally, the convolutional function of the memristor array requires the continuous sampling and calculation of multiple input blocks in a serial sliding manner. This approach cannot match the computational efficiency of fully connected structures and presents great challenges in achieving multi-value storage with high reliability. Precisely controlling of resistance states is a technical challenge for achieving the highly reliable multi-value storage. (4) The integration technology of memristor-based chips is currently incompatible with the existing information technology, presenting a significant challenge in achieving a higher level of integration architecture such as 3D chip. In summary, although the memristors hold great potential in development of brain-like neural networks, there are still substantial obstacles to overcome. Therefore, it is essential to focus on addressing current challenges and limitations, resolving issues related to the new architecture, and taking concrete steps toward promoting the future commercialization developments.

## Acknowledgments

This work is supported by the Hunan Science Fund for Distinguished Young Scholars (2023JJ10069), the 10.13039/501100001809National Natural Science Foundation of China (52172169), and the Project of State Key Laboratory of Precision Manufacturing for Extreme Service Performance, 10.13039/501100002822Central South University (ZZYJKT2024-02).

## Declaration of interests

There are no competing interests.
